# CVB-D attenuates experimental diabetic cardiomyopathy by alleviating mitochondrial dysfunction via the JAK1-STAT1 signaling axis in vivo and in vitro

**DOI:** 10.1186/s13020-026-01411-2

**Published:** 2026-05-13

**Authors:** Hang Su, Chun-qiang Zhang, Jiang-fei An, Yue-ting Tuo, Ting-ting Chen, Long Yang, Ling-yun Fu, Wen-bing Yao, Ling Tao, Yi-ni Xu, Xiang-chun Shen

**Affiliations:** 1 The State Key Laboratory of Discovery and Utilization of Functional Components in Traditional Chinese Medicine, Guizhou Medical University (Guizhou International Science and Technology Cooperation Base for Druggability Research of Natural Medicines), No. 6 Ankang Avenue, Guian New District, Guiyang, Guizhou, 561113 China; 2 The Department of Pharmacology of Materia Medica (The High Efficacy Application of Natural Medicinal Resources Engineering Center of Guizhou Province and The High Educational Key Laboratory of Guizhou Province for Natural Medicinal Pharmacology and Druggability), School of Pharmaceutical Sciences, Guizhou Medical University, No. 6 Ankang Avenue, Guian New District, Guiyang, Guizhou, 561113 China; 3 The Key Laboratory of Optimal Utilization of Natural Medicine Resources (The Union Key Laboratory of Guiyang City-Guizhou Medical University), School of Pharmaceutical Sciences, Guizhou Medical University, No. 6 Ankang Avenue, Guian New District, Guiyang, Guizhou, 561113 China; 4 Department of Pharmacy, Guiyang Maternal and Child Health Care Hospital, Guiyang, 550003 Guizhou China; 5 The Jiangsu Key Laboratory of Druggability of Biopharmaceuticals, School of Life Science and Technology, China Pharmaceutical University, No. 639 Longmian Dadao, Nanjing, 211198 Jiangsu China

**Keywords:** Diabetic cardiomyopathy, Cyclovirobuxine D, Mitochondrial function, Heart failure, JAK1-STAT1

## Abstract

**Background:**

Diabetic cardiomyopathy (DCM), as a prevalent cardiovascular complication in diabetes, involves cardiomyocyte dysfunction as a central pathological feature. Cyclovirobuxine D (CVB-D) is a naturally occurring bioactive alkaloid derived from *Buxus microphylla*. Emerging evidence suggests that CVB-D may ameliorate diabetes-associated cardiomyocyte failure. However, the protective effects of CVB-D against cardiomyocyte failure have not been extensively investigated, and the underlying molecular mechanisms remain unclear.

**Methods:**

A mouse model of diabetic cardiomyopathy was established by combining a high-fat diet (HFD) with streptozotocin (STZ). To examine the In vivo contribution of JAK1, AAV9 vectors targeting JAK1 were administered via tail-vein injection, with the corresponding negative-control vectors used in parallel. In vitro, a cardiomyocyte injury model was generated by exposing neonatal mouse ventricular myocytes (NMVMs) to palmitate and high-glucose (PA/HG) conditions. The cardioprotective effects of CVB-D were evaluated using Western blotting, flow cytometry, immunofluorescence microscopy, mitochondrial respiration assays, and ELISA-based measurements. Mechanistic investigations further integrated molecular docking, immunoprecipitation (IP), microscale thermophoresis (MST), surface plasmon resonance (SPR), and liquid chromatography-tandem mass spectrometry (LC-MS/MS) to define the molecular targets and JAK1/STAT1 signaling pathways underlying CVB-D activity.

**Results:**

CVB-D treatment robustly improved mitochondrial dysfunction in Diabetic cardiomyopathy and attenuated heart failure-like phenotypes in cardiomyocytes both in vivo and in vitro. Mechanistically, CVB-D reduced JAK1 expression and concomitantly diminished STAT1 phosphorylation, thereby alleviating cardiomyocyte injury. Moreover, convergent evidence from IP, MST, and SPR assays supported a central role for the JAK1-STAT1 axis in mediating the functional effects of CVB-D. LC-MS/MS analysis further identified STAT1 residues T598 and T699 as putative JAK1-dependent phosphorylation regulatory sites in NMVMs. Consistently, genetic knockdown or pharmacological inhibition of JAK1 improved DCM-related phenotypes, whereas enforced JAK1 expression or pharmacological activation blunted the protective effects of CVB-D, indicating that CVB-D-mediated cardioprotection requires suppression of JAK1-STAT1 signaling.

**Conclusion:**

Our findings indicate that CVB-D enhances mitochondrial function by suppressing the JAK1-STAT1 signaling axis, thereby ameliorating heart failure associated with DCM. These results suggest that CVB-D may represent a promising therapeutic candidate for the treatment of DCM-related heart failure.

**Graphical abstract:**

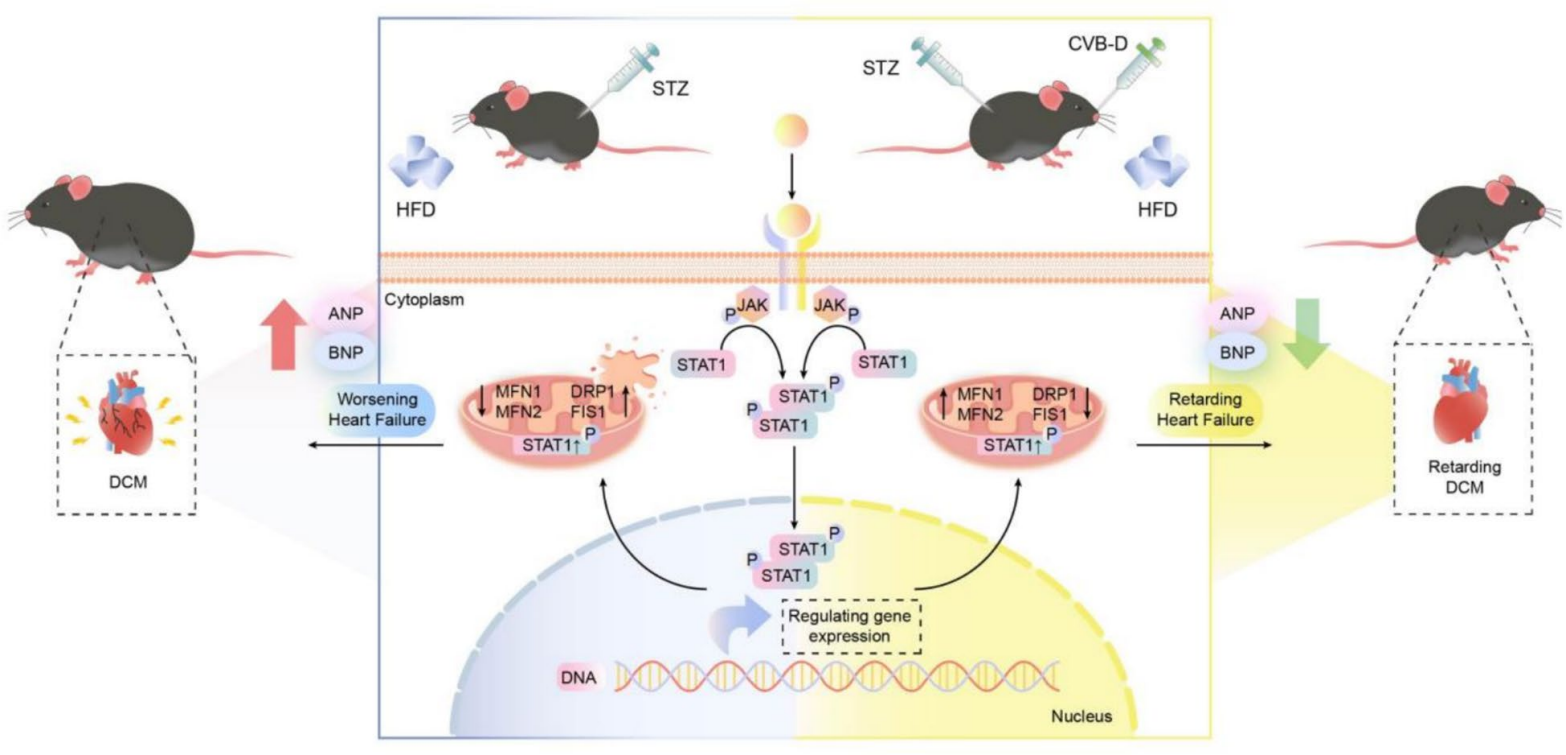

**Supplementary Information:**

The online version contains supplementary material available at 10.1186/s13020-026-01411-2.

## Introduction

Diabetes mellitus (DM) is broadly classified into type 1 (T1DM) and type 2 (T2DM), with T2DM accounting for > 90% of cases worldwide. Importantly, T2DM are associated with an increased risk of developing diabetic cardiomyopathy (DCM) and progression to heart failure (HF) [1].

There is currently no universally accepted definition of diabetic cardiomyopathy. In general, DCM describes diabetes-associated myocardial dysfunction that occurs independent of other major cardiac comorbidities, such as structural heart disease, coronary artery disease, or hypertension [2]. DCM is typically characterized by left ventricular dilatation, cardiomyocyte hypertrophy, and interstitial myocardial fibrosis, which collectively contribute to systolic and/or diastolic dysfunction and may ultimately progress to HF [3]. Mitochondrial dysfunction, excessive oxidative stress, inflammatory activation, and dysregulated calcium homeostasis are central contributors to the pathogenesis of DCM, promoting cardiomyocyte apoptosis and ultimately impairing cardiac function [4, 5].

Mitochondria are essential cellular organelles that serve as the primary sites of energy production. By coupling electron transport to oxidative phosphorylation, mitochondria generate adenosine triphosphate (ATP), thereby supplying energy to support normal cellular functions [6]. Mitochondrial dynamics encompasses the coordinated processes of fusion and fission [7]. In mammalian cells, mitochondrial fusion is primarily regulated by mitofusin 1 and mitofusin 2 (MFN1/2) [8]. Mitochondrial fission is primarily mediated by dynamin-related protein 1 (DRP1) and the outer-membrane adaptor fission protein 1 (FIS1) [9]. Under conditions of chronic hyperglycemia and insulin resistance, mitochondrial metabolism and quality control in cardiomyocytes are frequently disturbed. This dysfunction reduces ATP generation and enhances reactive oxygen species (ROS) production, while also perturbing intracellular calcium homeostasis [10]. Collectively, these alterations compromise cardiomyocyte viability and contractile performance, thereby promoting maladaptive remodeling and progressive deterioration of cardiac structure and function, which can ultimately culminate in HF [11, 12]. In HF, circulating natriuretic peptides-particularly atrial natriuretic peptide (ANP) and the endogenously produced B-type natriuretic peptide (BNP)-are typically elevated and are widely used as biomarkers reflecting myocardial stress and heart failure severity [13, 14].

Janus kinases (JAKs) are non-receptor tyrosine kinases essential for cytokine signaling [15]. The four family members-JAK1, JAK2, JAK3, and TYK2-are constitutively bound to cytokine receptors [16]. Upon cytokine binding, JAKs activate and phosphorylate STAT proteins, initiating the JAK-STAT pathway, which plays a key role in the pathogenesis and progression of HF [17]. STATs (Signal Transducers and Activators of Transcription) are a family of proteins that play a key role within cells [18]. STAT1 is a member of this family, which comprises seven transcription factors (STAT1, STAT2, STAT3, STAT4, STAT5A, STAT5B, and STAT6) that collectively mediate cellular responses to various cytokines and growth factors [19]. The JAK1-STAT1 signaling pathway influences mitochondrial function by regulating energy metabolism, and mitochondrial apoptotic pathways. Research indicates that STAT1 activation can suppress the mitochondrial electron transport chain and promote glycolysis, thereby altering cellular energy metabolism patterns [20]. Additionally, this pathway increases mitochondrial ROS production, forming a positive feedback loop of oxidative stress [21]. In neurodegenerative and cardiovascular diseases, abnormal activation of the JAK1-STAT1 pathway disrupts mitochondrial homeostasis and exacerbates cellular dysfunction [22]. However, it remains unclear whether the JAK1-STAT1 signaling axis participates in the pathogenesis of HF, and whether it represents a viable therapeutic target.

Cyclovirobuxine D (CVB-D), a bioactive alkaloid mainly isolated from the traditional Chinese medicinal herb *Buxus sinica*, has been reported to exert protective effects in cardiovascular and cerebrovascular disorders, including cardioprotection and attenuation of ischemia-related injury [17]. In addition, CVB-D has been reported to suppress LPS-induced inflammatory signaling in RAW264.7 macrophages, largely through inhibition of the JAK-STAT pathway [23, 24]. However, the effect of CVB-D on delaying the progression of heart failure has not been extensively studied. This study employs both in vivo and in vitro experiments to investigate how CVB-D alleviates mitochondrial dysfunction and delays HF through the JAK1-STAT1 signaling axis.

## Materials and methods

### Reagents

Cyclovirobuxine D (CVB-D; C₂₆H₄₆N₂O) was purchased from Chengdu Puxi Biotechnology Co., Ltd. (Cat. No. PS3166; Lot No. PS020930) with a purity of 98%. The high-fat diet (HFD; 60 kcal% fat) was obtained from Moldiets Biotechnology (Cat. No. M10160). The JAK1-Mus-3306 construct was purchased from Shanghai GenePharma Co., Ltd., with the following sequences: sense strand, 5′-cuguccugaugagguuuautt-3′, and antisense strand, 5′-auaaaccucaucaggacagtt-3′. The adeno-associated virus serotype 9 (AAV9) vector expressing JAK1 shRNA (AAV9-U6-JAK1; 5′-cuguccugaugagguuuautt-3′) was also purchased from Shanghai GenePharma Co., Ltd.

### Animals

Male C57BL/6J mice (6–8 weeks old; 18–24 g) were purchased from the Experimental Animal Center of Guizhou Medical University. After a 1-week acclimation period, mice were randomly assigned to either a normal diet group (control) or a high-fat diet (HFD) group. To induce DCM, HFD-fed mice received intraperitoneal injections of streptozotocin (STZ; 45 mg/kg/day) for four consecutive days. Mice with fasting blood glucose levels ≥ 11.1 mmol/L were considered diabetic and included in subsequent experiments. Diabetic mice were then randomly and evenly allocated into four groups: DCM, DCM plus low-dose CVB-D (0.5 mg/kg/day; CVB-D-L), DCM plus high-dose CVB-D (1 mg/kg/day; CVB-D-H), and DCM plus metformin (250 mg/kg/day; Met). CVB-D and metformin were administered once daily by oral gavage for 8 weeks, while mice in the control and DCM groups received an equal volume of saline. At the end of the treatment period, mice were euthanized under isoflurane anesthesia for subsequent analyses.

### Echocardiographic function

Mice were anesthetized by inhalation of 1–2% isoflurane and placed on a heated platform, with continuous low-dose anesthesia maintained throughout the procedure. Cardiac function was assessed by M-mode echocardiography using a VINNO6LAB ultrasound system (VINNO, Suzhou, China) equipped with a 10-MHz transducer. All measurements were performed by an operator blinded to group allocation.

### Ultrastructural examination of the myocardium

Fresh left ventricular tissue was cut into ~ 1 mm^3^ pieces, pre-fixed in 2.5% glutaraldehyde, and then post-fixed in 1% osmium tetroxide. Samples were dehydrated through a graded acetone series, followed by stepwise infiltration with acetone and Epon-812 resin mixtures at ratios of 3:1, 1:1, and 1:3, and finally embedded in pure Epon-812. Ultrathin Sections (60–90 nm) were prepared using an ultramicrotome and collected onto copper grids. Sections were stained at room temperature with uranyl acetate for 10–15 min followed by lead citrate for 1–2 min. The stained sections were examined and imaged using a transmission electron microscope (JEM-1400 Flash).

### Separation and purification of primary mouse cardiomyocytes

Specific pathogen-free (SPF) C57BL/6J mice (18–22 g) were purchased from Beijing Biotechnology Co., Ltd. Primary neonatal mouse ventricular myocytes (NMVMs) were isolated and purified from 1 to 3-day-old neonatal mice and cultured in DMEM at 37 °C in a humidified incubator with 5% CO₂. Cardiomyocytes (1 × 10^5^ cells/mL) were treated with 100 μM 5-bromo-2′-deoxyuridine (5-BrdU) to inhibit the proliferation of cardiac fibroblasts.

### Western blotting analysis

Cardiac tissues and NMVMs were washed three times with pre-chilled PBS and then lysed for protein extraction. Protein concentrations were determined using a BCA assay kit, and samples were denatured prior to SDS-PAGE. Proteins were separated by SDS-PAGE and transferred onto PVDF membranes. Membranes were blocked with 5% (w/v) non-fat milk at room temperature for 1.5 h and then incubated overnight at 4 °C with primary antibodies against p-JAK1, JAK1, STAT1, MFN1, MFN2, DRP1, FIS1, ANP, BNP, and β-actin. After washing, membranes were incubated with appropriate secondary antibodies for 1.5 h at room temperature. Immunoreactive bands were visualized using the NcmECL High detection kit (NCM Biotechnology, Suzhou, China). Protein levels were normalized to β-actin, and band intensities were quantified using Image Lab software (Bio-Rad, v5.2) and ImageJ (NIH, v1.52a).

### Mitochondrial membrane potential (ΔΨm) assay

A JC-1 staining kit (Cat. No. M8650; Solarbio, Beijing, China) was used to assess mitochondrial membrane potential (ΔΨm). After drug treatment, primary neonatal mouse ventricular myocytes (NMVMs) were washed and incubated with JC-1 working solution for 20 min at 37 °C. Cells were then washed three times with JC-1 staining buffer. Fluorescence images were captured using a Nikon Eclipse C1 microscope (Tokyo, Japan), and ΔΨm was quantified using ImageJ software.

### Network pharmacology analysis

Target prediction for CVB-D. The two-dimensional (2D) and three-dimensional (3D) chemical structures of cyclovirobuxine D (CVB-D) were obtained from the PubChem database (http://www.ncbi.nlm.nih.gov/home/chemicals). Putative therapeutic targets of CVB-D were identified using SwissTargetPrediction (http://www.swisstargetprediction.ch/), PharmMapper (https://www.lilab-ecust.cn/pharmmapper/), and the Comparative Toxicogenomics Database (CTD) (https://ctdbase.ord/). For SwissTargetPrediction, protein targets and functional annotations were predicted based on the PubChem SMILES string (https://pubchem.ncbi.nlm.nih.gov/); the screening criteria were restricted to *Homo sapiens* and probability > 0. PharmMapper integrates multiple pharmacophore databases to identify potential targets algorithmically; after uploading the CVB-D SDF file, up to 300 pharmacophore models were selected, and predicted targets with a Norm Fit score > 0.5 were retained. In CTD, CVB-D–related targets were retrieved, and those with an interaction count > 20 were considered valid targets. After removing redundant entries across the three databases, a non-redundant set of candidate CVB-D targets was obtained.

Identification of disease-related targets.Potential diabetic cardiomyopathy (DCM)–associated targets were collected from GeneCards (https://www.genecards.org/), CTD (https://ctdbase.org), and the Online Mendelian Inheritance in Man (OMIM) database (https://www.omim.org). All targets were merged and deduplicated to generate the DCM-related target set. Venny 2.1 (https://bioinfogp.cnb.csic.es/tools/venny/) was used to visualize the overlap between CVB-D targets and DCM targets, and the intersecting genes were defined as candidate targets potentially mediating the therapeutic effects of CVB-D against DCM.

Protein–protein interaction (PPI) network and hub target screening.The intersecting targets were imported into the STRING database (http://string-db.org/) to construct a protein–protein interaction (PPI) network, with the organism set to *Homo sapiens* and the confidence score threshold set to 0.7. The PPI data exported from STRING were further analyzed in Cytoscape (v3.10.0) to identify hub targets based on topological parameters including degree, closeness centrality, and betweenness centrality, and a hub-target interaction network was generated.

GO and KEGG enrichment analyses.Candidate targets of CVB-D against DCM were submitted to the DAVID platform (http://david.nifcrf.gov/) for Gene Ontology (GO) and Kyoto Encyclopedia of Genes and Genomes (KEGG) pathway enrichment analyses, with *Homo sapiens* selected as the background. The screening criteria were set as a minimum count of 3, *p* < 0.05, and a minimum enrichment score of 1.5. Enrichment results for biological process (BP), cellular component (CC), molecular function (MF), and KEGG pathways were collected and visualized using an online bioinformatics platform (https://www.bioinformatics.com.cn) to generate GO bubble plots and KEGG pathway plots.

Drug–disease–target–pathway network construction.To systematically interpret the potential molecular mechanisms of CVB-D against DCM, the hub targets and the top 20 KEGG pathways were imported into Cytoscape (v3.10.0) to construct a drug–disease–target–pathway network.

### Measurement of oxygen consumption rate (OCR)

Mitochondrial function in treated NMVMs was assessed by measuring the oxygen consumption rate (OCR) using an Oroboros O2k respirometer. Following baseline respiration recording, mitochondrial modulators were sequentially added-oligomycin (1 μM), carbonyl cyanide 4-(trifluoromethoxy) phenylhydrazone (FCCP; 2 μM), and rotenone/antimycin A (1 μM each)-to determine ATP-linked respiration, maximal respiration, and spare respiratory capacity, respectively.

### Detection of creatine kinase isoenzyme (CK-MB) (ELISA assay)

Reagents, samples, and standards were prepared according to the manufacturer’s instructions. Briefly, 50 μL of prepared samples or standards and 50 μL of biotinylated antigen working solution were added to each well and incubated at 37 °C for 30 min. The plate was washed five times, followed by addition of 50 μL of streptavidin-HRP working solution and incubation at 37 °C for 10 min. Then, 50 μL of stop solution was added to terminate the reaction. Absorbance (OD) was measured at 450 nm using a microplate reader within 10 min.

### Immunofluorescence analysis

Paraffin sections were deparaffinized in xylene and rehydrated through a graded ethanol series to water. Antigen retrieval was performed using citrate or EDTA buffer, followed by cooling and washing three times with PBS. Sections were permeabilized with Triton X-100 for 30 min and blocked with 1% BSA for 30 min, then incubated with primary antibodies overnight at 4 °C. After PBS washes, sections were incubated with the corresponding secondary antibodies for 50 min at room temperature in the dark, followed by DAPI counterstaining for 10 min. Autofluorescence was quenched for 5 min and rinsed, and sections were mounted with an anti-fade medium and stored at 4 °C in the dark. Images were acquired using a Nikon Eclipse C1 fluorescence microscope or an Olympus FV1000 confocal microscope and quantified with ImageJ.

### Microscale thermophoresis (MST)

Microscale thermophoresis (MST) is an emerging technique for analyzing biomolecular interactions based on the principle of thermophoresis. MST was used to determine the binding affinity between JAK1 and STAT1, and the data were analyzed using a NanoTemper Technologies affinity analyzer to obtain the equilibrium dissociation constant (KD).

### Surface plasmon resonance (SPR) binding assay

Surface plasmon resonance (SPR) was employed to evaluate the interaction between mouse JAK1 and STAT1 proteins. A freshly prepared mixture of 400 mM EDC and 100 mM NHS was used for amine coupling. Ligand immobilization was performed on a CM5 sensor chip at a flow rate of 10 μL/min for 420 s. After immobilization, analyte binding was assessed using a multi-cycle kinetic assay. JAK1 protein was diluted in the running buffer to generate eight concentrations ranging from 0.00 to 0.03 μM for kinetic analysis. Recombinant JAK1 (Cat. No. WX03D3CD) and STAT1 (Cat. No. WXOOB1C7) proteins, each with a purity > 80%, were used in the SPR experiments.

### LC-MS/MS analysis

The analysis was performed using a hybrid ion-trap Orbitrap liquid chromatography-tandem mass spectrometry system (Orbitrap Elite LC-MS/MS; Thermo Fisher Scientific, USA). Target bands were excised from gel strips, followed by protein extraction, desalting, lyophilization, and LC-MS/MS analysis. Raw MS data were processed using MaxQuant (v2.1) for database searching, peptide/protein identification, and downstream analysis. The UniProt STAT1.fasta database was used for the searches.

### Statistical analysis

All the data were subjected to analysis via GraphPad Prism 8.0 software. One-way ANOVA was used for multigroup comparisons, whereas Student’s *t* test was used for comparisons between two groups. All statistically analyzed outcomes are presented as the mean ± SEM. A *p* value < 0.05 indicates a statistically significant difference in this study.

## Results

### CVB-D improves cardiac dysfunction in mice with HFD-induced DCM

**Fig. 1:**
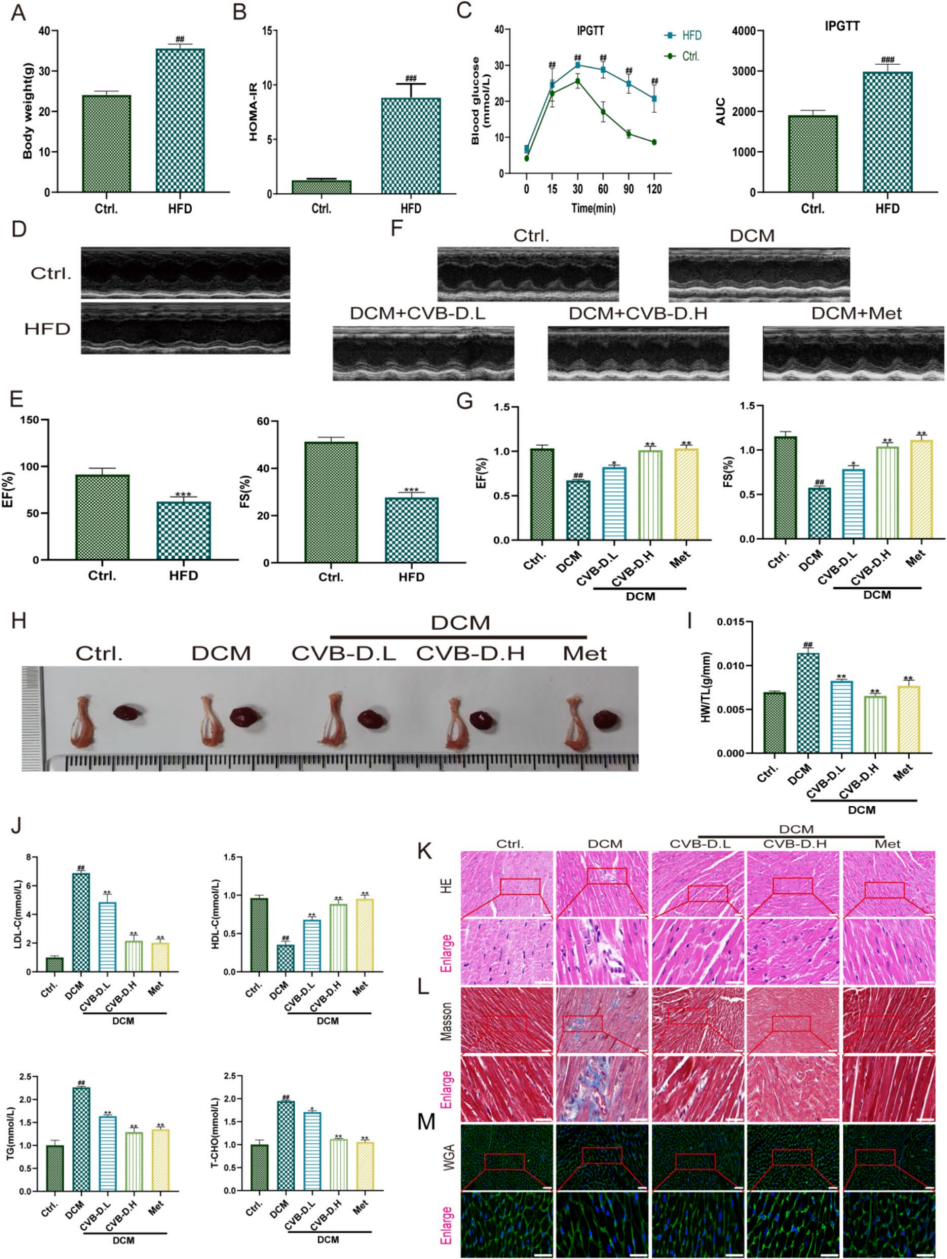
CVB-D improves cardiac dysfunction in mice with HFD-induced diabetic cardiomyopathy (DCM). **A** Body weight of mice (n = 6). **B** Insulin resistance index in mice (n = 6). **C** Oral glucose tolerance test (OGTT) results and the area under the curve (AUC) of blood glucose in mice (n = 6). **D**–**G** Representative M-mode echocardiographic images and quantitative analysis of ejection fraction (EF%) and fractional shortening (FS%) (n = 6). **H** Representative gross anatomical images of mouse hearts and tibiae (n = 6). **I** Heart weight-to-tibia length ratio (HW/TL) (n = 6). **J** CVB-D on the serum levels of low-density lipoprotein (LDL), high-density lipoprotein (HDL), triglycerides (TG) and total cholesterol (TC) (n > 6). **K** Hematoxylin and eosin (H&E) staining of heart tissues (scale bar = 50 μm). **L** Masson’s trichrome staining showing collagen deposition in heart tissues (scale bar = 50 μm). **M** Wheat germ agglutinin (WGA) staining of cardiomyocytes from DCM mice (scale bar = 50 μm). ^#^*p* < 0.05, ^##^*p* < 0.01 versus the control group; ^*^p < 0.05, ^**^p < 0.01 versus the model group

A mouse model of DCM was established by first feeding a high-fat diet to induce insulin resistance, followed by low-dose intraperitoneal injections of streptozotocin to induce moderate hyperglycemia, thereby mimicking the pathogenesis of T2DM. After 12 weeks, HFD-treated mice exhibited significantly increased body weight and pronounced insulin resistance compared with control mice (Fig. 1A, B). In agreement with these metabolic alterations, intraperitoneal glucose tolerance testing (IPGTT) showed markedly elevated blood glucose levels and a significantly increased area under the curve (AUC) in the HFD group, indicating impaired glucose tolerance (Fig. 1C). Notably, CVB-D treatment did not reduce blood glucose levels during the intervention period (Fig. S1A), suggesting that the subsequent cardioprotective effects of CVB-D are largely independent of systemic glycemic improvement.

Cardiac function was assessed by echocardiography, which confirmed the successful establishment of the DCM phenotype in mice. Compared with controls, HFD-induced mice displayed significant left ventricular systolic dysfunction, as evidenced by decreased ejection fraction (EF%) and fractional shortening (FS%) (Fig. 1D, E). Mice were subsequently treated by oral gavage with CVB-D at two doses (0.5 or 1 mg/kg/day) or metformin (Met; 250 mg/kg/day). Relative to untreated DCM mice, both CVB-D and metformin significantly improved cardiac contractile performance, reflected by increased EF% and FS% (Fig. 1F, G). Consistent with these functional improvements, the heart weight-to-tibia length ratio (HW/TL) was elevated in DCM mice and was significantly reduced following CVB-D treatment (Fig. 1H, I).

In addition to improving cardiac function, CVB-D ameliorated DCM-associated metabolic abnormalities. Serum lipid profiling revealed decreased high-density lipoprotein (HDL) levels and increased triglyceride (TG), total cholesterol (TC), and low-density lipoprotein (LDL) levels in DCM mice; these dyslipidemic alterations were significantly reversed by CVB-D administration (Fig. 1J). Histopathological analyses corroborated these findings. Hematoxylin–eosin (H&E) and wheat germ agglutinin (WGA) staining demonstrated enlarged cardiomyocyte nuclei, cardiomyocyte hypertrophy, and disorganized myocardial architecture in DCM mice, all of which were substantially alleviated by CVB-D (Fig. 1K, M). Moreover, Masson’s trichrome staining revealed markedly increased myocardial collagen deposition in DCM mice, whereas CVB-D treatment significantly reduced myocardial fibrosis (Fig. 1L). Collectively, these data indicate that CVB-D effectively attenuates pathological cardiac remodeling and improves systolic dysfunction in DCM, thereby delaying disease progression in vivo.

### Network pharmacology suggests that CVB-D may protect against DCM via STAT1-associated signaling

**Fig. 2:**
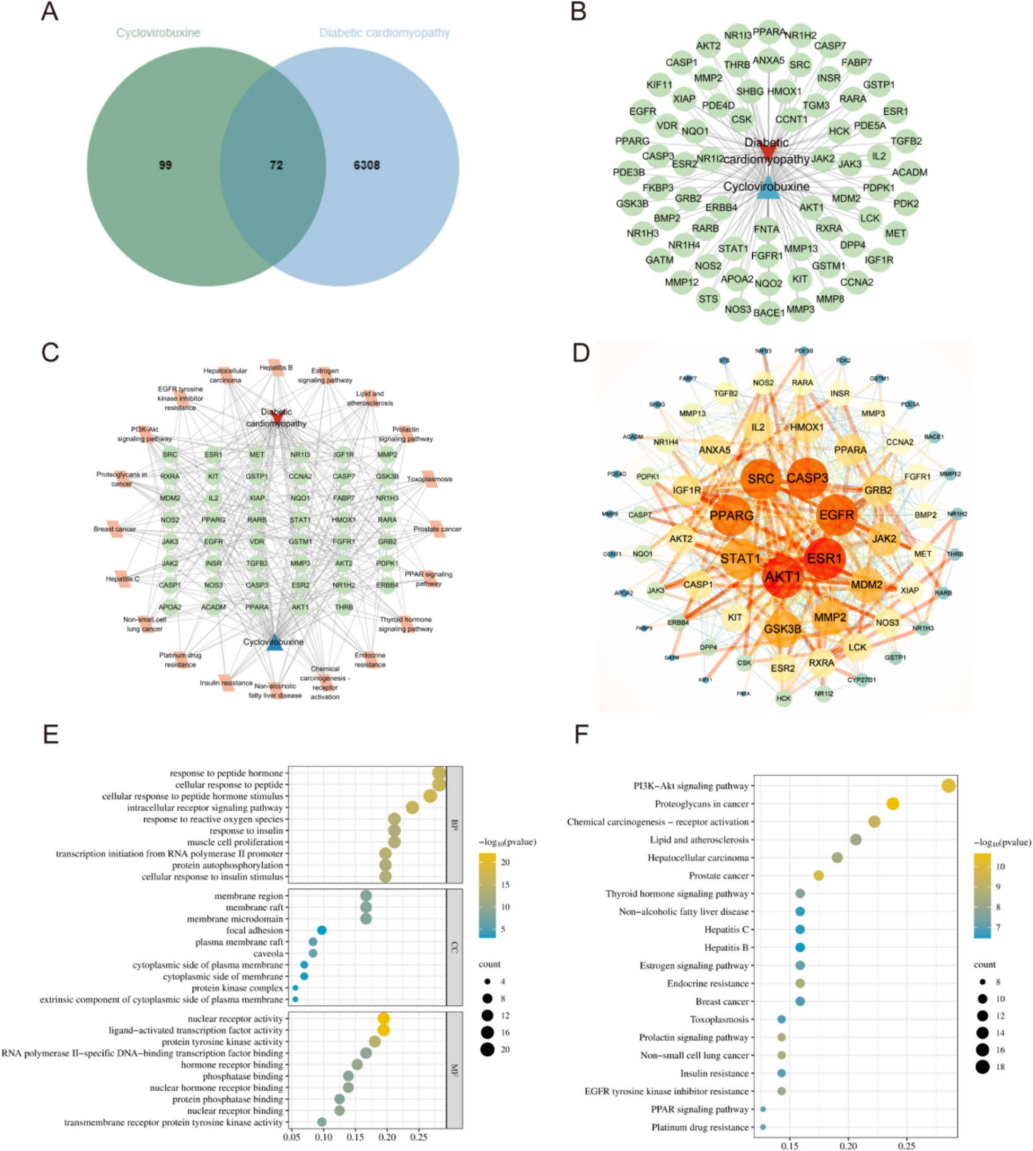
Network pharmacology suggests that CVB-D may protect against DCM via STAT1-associated signaling. **A**, **B** Venn analysis was performed between predicted CVB-D targets and DCM-associated genes, identifying 72 overlapping targets. **C** A CVB-D–DCM intersecting-target network was constructed and visualized. **D** Protein–protein interaction (PPI) analysis was conducted for the intersecting targets, and hub nodes were screened based on network topology parameters. **E** Gene Ontology (GO) enrichment analysis was performed for the intersecting targets. **F** KEGG pathway enrichment analysis was performed for the intersecting targets (*P* < 0.05)

A total of 171 putative targets of CVB-D and 6,380 DCM-related genes were retrieved from public databases. Venn analysis identified 72 overlapping targets, suggesting that CVB-D may modulate DCM through a multi-target mode of action (Fig. 2A, B). Network construction and PPI analysis further revealed a highly interactive core module among the intersecting targets. Topological analysis highlighted AKT1, EGFR, STAT1, ESR1, SRC, PPARG, IGF1R, MMP2, CASP3, and GSK3B as central hub nodes, implying pivotal regulatory roles in the CVB-D-DCM network (Fig. 2C, D).

Gene Ontology (GO) term analysis across biological process (BP), cellular component (CC), and molecular function (MF) categories reveals that CVB-D intervention in DCM operates through a coordinated regulatory framework: receptor-mediated signaling initiates kinase phosphorylation cascades, culminating in transcriptional control (Fig. 2E).

KEGG pathway enrichment analysis further showed that the 72 intersecting targets were significantly enriched in multiple pathways linked to kinase signaling and metabolic stress (*P* < 0.05), with the PI3K-Akt signaling pathway among the most prominent. Additional enriched pathways included insulin resistance, the PPAR signaling pathway, endocrine resistance, and EGFR tyrosine kinase inhibitor resistance (Fig. 2F). Integrating the GO/KEGG enrichment patterns with network topology, we prioritized STAT1 for subsequent mechanistic validation. As a prototypical phosphorylation-dependent transcription factor hub, STAT1 integrates upstream kinase inputs and drives downstream transcriptional reprogramming, consistent with the enrichment signature of kinase-driven transcriptional responses and metabolism-related stress pathways (Fig. 2E, F). Thus, network pharmacology supports STAT1 as a key candidate target mediating CVB-D effects in DCM and provides a systems-level rationale for further investigation of its upstream regulator JAK1 and associated phosphorylation events.

### CVB-D improves mitochondrial function in DCM mice by restoring mitochondrial homeostasis

**Fig. 3:**
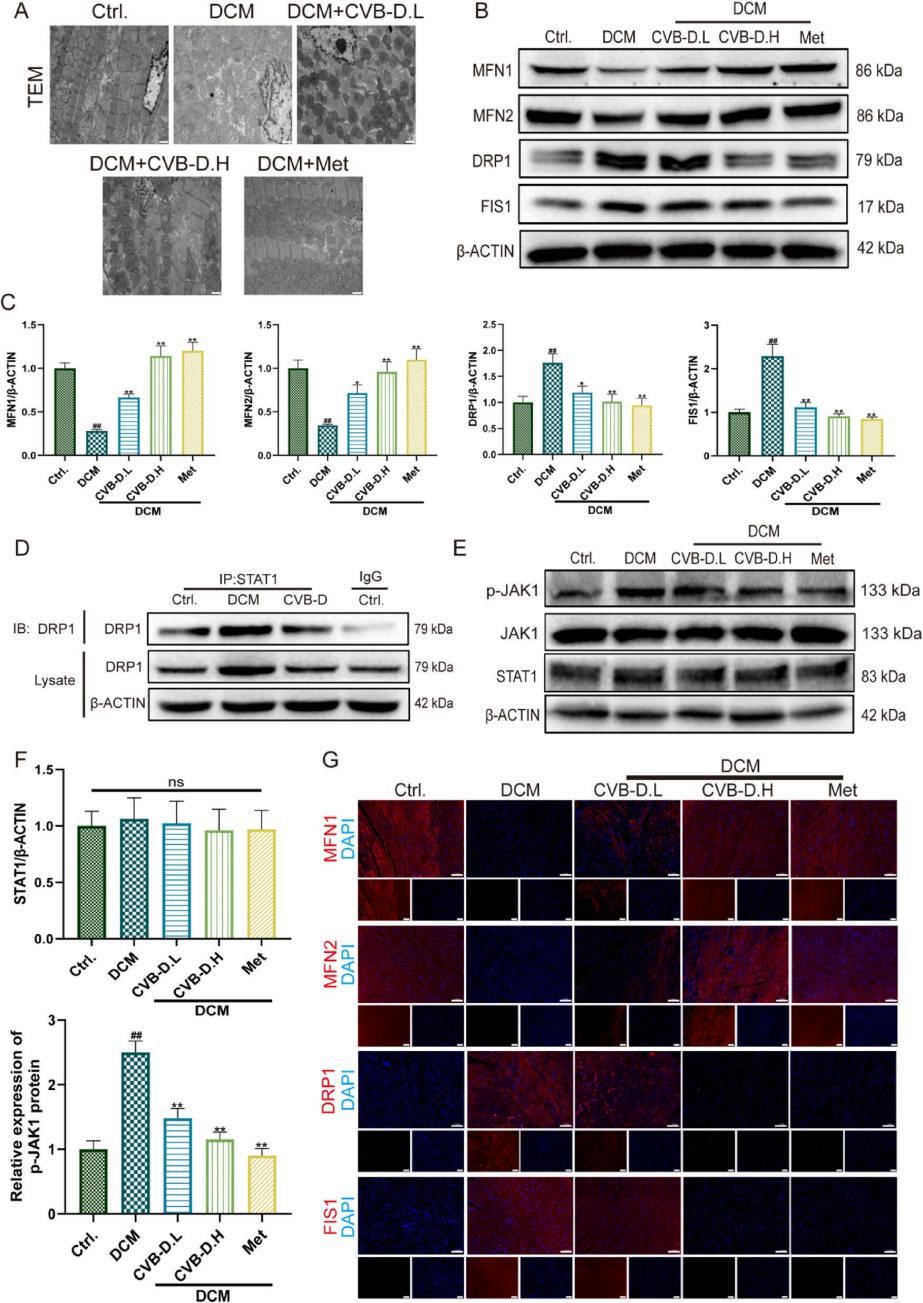
CVB-D improves mitochondrial function in DCM mice by restoring mitochondrial homeostasis. **A** Mitochondrial ultrastructure in myocardial tissues from different groups, shown as representative transmission electron microscopy (TEM) images (scale bar = 2 µm). **B**, **C** Densitometric quantification of mitochondrial fusion-related proteins (MFN1 and MFN2) and fission-related proteins (DRP1 and FIS1) in mouse cardiac tissues (n = 5). **D** Immunoprecipitation (IP) assays showing the interaction between STAT1 and DRP1 in cardiac lysates. **E** Representative western blots of p-JAK1, total JAK1, and STAT1. **F** Densitometric analysis of STAT1 expression and p-JAK1 levels based on western blotting (n = 5). **G** Representative immunofluorescence images of MFN1, MFN2, DRP1, and FIS1 in cardiac tissues (scale bar = 100 µm). ^#^*p* < 0.05, ^##^*p* < 0.01 versus the control group; ^*^p < 0.05, ^**^p < 0.01 versus the model (DCM) group; ns, not significant

To assess the damage to mitochondria within the cardiomyocytes of DCM mice, we conducted ultrastructural observation by transmission electron microscopy (TEM). TEM revealed pronounced mitochondrial fragmentation and disorganization of cristae architecture in the myocardium of DCM mice, whereas CVB-D treatment partially normalized mitochondrial morphology and improved cristae integrity (Fig. 3A). Consistent with these ultrastructural changes, myocardial ATP content was significantly decreased in DCM mice and was substantially restored following CVB-D administration (Fig. S2A). In parallel, the elevated L-lactate dehydrogenase (L-LDH) activity observed in DCM mice was significantly reduced by CVB-D treatment (Fig. S2B). To determine whether CVB-D modulates mitochondrial dynamics, we evaluated key regulators of mitochondrial fusion and fission. Western blot analysis demonstrated downregulation of the mitochondrial fusion proteins MFN1 and MFN2 in DCM hearts, accompanied by upregulation of the fission-related proteins DRP1 and FIS1; notably, these alterations were reversed by CVB-D (Fig. 3B, C). Immunofluorescence (IF) staining further corroborated these findings, showing restoration of MFN1/MFN2 signals and a reduction in DRP1/FIS1 expression in CVB-D-treated DCM hearts (Fig. 3G). Prior studies have implicated STAT1 signaling in the regulation of DRP1-dependent mitochondrial dynamics. For example, Zhang J. et al. reported that NSD2 overexpression facilitates STAT1 nuclear translocation and enhances STAT1 phosphorylation, which is associated with increased mitochondrial fragmentation [25]. In addition, Zhen C. et al. demonstrated that STAT1 can regulate mitochondrial function through a p-Drp1-dependent pathway [26]. In agreement with these observations, immunoprecipitation assays confirmed an association between STAT1 and DRP1 in DCM myocardium (Fig. 3D). Moreover, given evidence that CVB-D suppresses activation of the JAK-STAT pathway, we examined JAK1/STAT1 signaling and found that total JAK1 and STAT1 protein levels were unchanged, while phosphorylated JAK1 (p-JAK1) was markedly increased in DCM mice and was significantly attenuated by CVB-D treatment (Fig. 3E, F). Collectively, these results indicate that CVB-D alleviates mitochondrial structural damage and functional impairment in diabetic hearts, at least in part, by restoring the balance between mitochondrial fusion and fission and by suppressing aberrant JAK1 activation in the context of STAT1-DRP1 interaction.

### CVB-D alleviates HG/PA-induced injury in primary mouse cardiomyocytes by restoring mitochondrial homeostasis

**Fig. 4:**
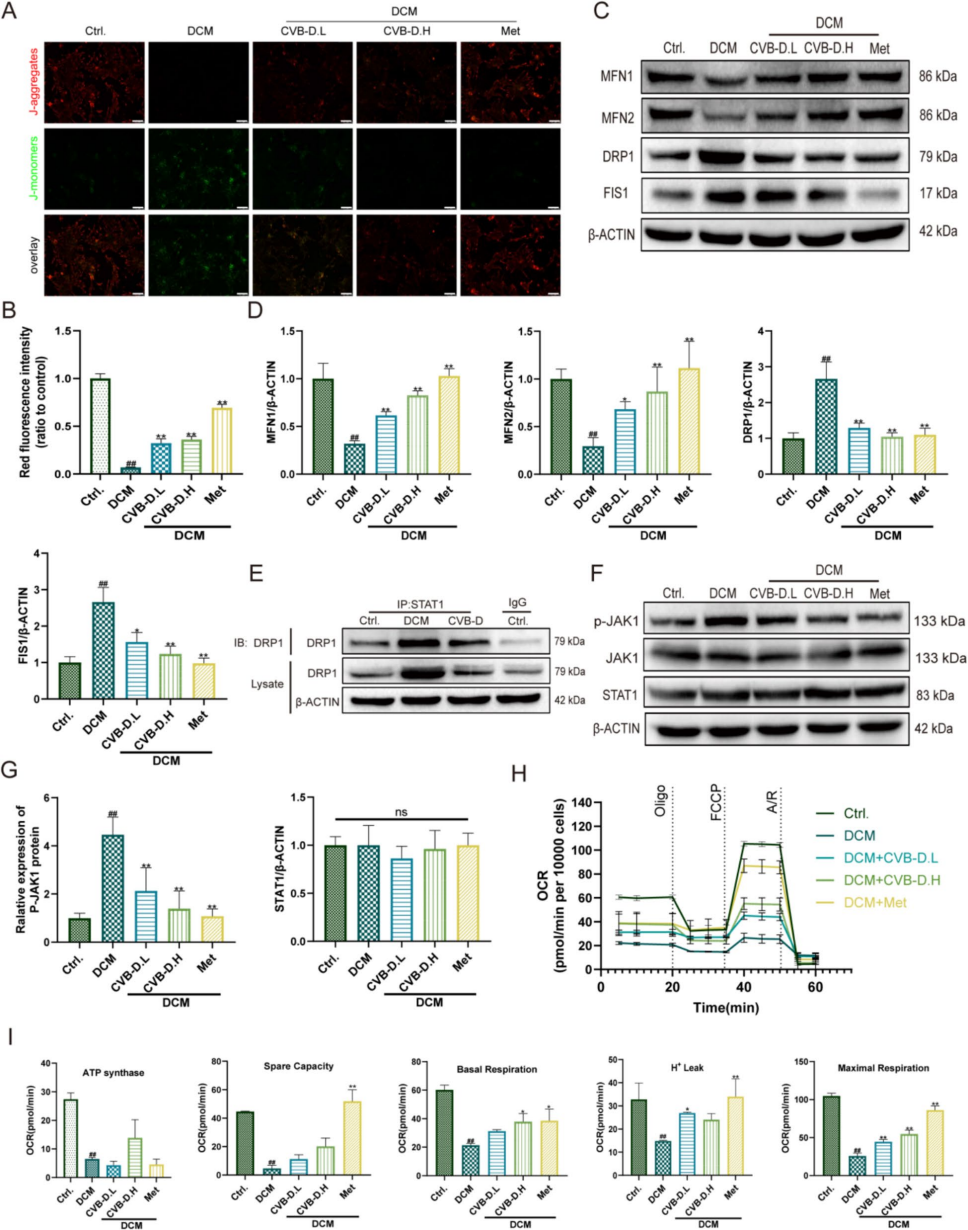
CVB-D alleviates HG/PA-induced injury in primary mouse cardiomyocytes by restoring mitochondrial homeostasis. **A** Representative JC-1 staining images showing changes in mitochondrial membrane potential in NMVMs from different groups (scale bar = 50 µm). **B** Quantification of JC-1 red fluorescence intensity using ImageJ (n = 5). **C**, **D** Western blot analysis of the mitochondrial fusion proteins MFN1 and MFN2 and the fission-related proteins DRP1 and FIS1 in NMVMs, with densitometric quantification (n = 5). **E** Immunoprecipitation (IP) assays validating the interaction between STAT1 and DRP1 in cardiomyocyte lysates (n = 3). **F**, **G** Western blot analysis of p-JAK1, total JAK1, and STAT1 protein levels in NMVMs, with quantitative analysis (n = 5). **H**, **I** Oxygen consumption rate (OCR) was measured using the Oroboros O2K assay to assess mitochondrial respiratory function (n = 3). ^#^*p* < 0.05, ^##^*p* < 0.01 versus the control group; ^*^p < 0.05, ^**^p < 0.01 versus the model group; ns, not significant

Neonatal mouse ventricular myocytes (NMVMs) were successfully isolated and verified by immunofluorescence staining for cardiac troponin T (cTnT) (Fig. S3A). To establish an in vitro model of DCM injury, NMVMs were challenged with HG/PA. Exposure to 40 mM glucose and 200 μM palmitate for 48 h markedly reduced cell viability (Fig. S3B). Pretreatment with CVB-D significantly improved NMVM viability at effective concentrations (0.05–0.1 μM) (Fig. S3C), accompanied by reduced L-lactate dehydrogenase (L-LDH) release (Fig. S3D) and restoration of intracellular ATP content (Fig. S3E). In addition, HG/PA induced excessive ROS accumulation, which was substantially attenuated by CVB-D (Fig. S3F). Mitochondrial oxidative stress levels in NMVMs were assessed using Mito-ROS staining. The results demonstrated that DCM-induced NMVMs exhibited significantly increased ROS compared to the control group. Treatment with CVB-D markedly improved these abnormalities (Fig. S3G). JC-1 staining further revealed a pronounced loss of mitochondrial membrane potential (ΔΨm) in HG/PA-treated NMVMs, as evidenced by decreased red fluorescence and increased green fluorescence; these changes were reversed by CVB-D pretreatment (Fig. 4A, B).

Mechanistically, western blot analysis indicated that HG/PA disrupted mitochondrial dynamics, characterized by downregulation of the mitochondrial fusion proteins MFN1 and MFN2 and upregulation of the fission-related proteins DRP1 and FIS1; CVB-D effectively reversed these alterations (Fig. 4C, D). Consistent with the in vivo findings, immunoprecipitation assays confirmed an interaction between STAT1 and DRP1 in primary cardiomyocytes (Fig. 4E). Moreover, although total JAK1 and STAT1 protein levels were not significantly altered, p-JAK1 was markedly increased in HG/PA-treated NMVMs and was significantly suppressed by CVB-D (Fig. 4F, G). qRT‒PCR analysis confirmed no significant difference in STAT1 mRNA levels (Fig. S3H). To further evaluate cellular respiration and mitochondrial function, we employed the Oroboros O2k high-resolution respirometer. The assessment revealed that HG/PA exposure impaired mitochondrial bioenergetics, leading to reductions in ATP-linked respiration, maximal respiration, proton leak, and spare respiratory capacity. These deficits were significantly mitigated by CVB-D treatment (Fig. 4H, I). Collectively, these results indicate that CVB-D protects NMVMs against HG/PA-induced injury and mitochondrial dysfunction, at least in part, by restoring the balance between mitochondrial fusion and fission and by inhibiting aberrant JAK1-STAT1-DRP1 signaling.

### CVB-D attenuates heart failure progression in diabetic dilated cardiomyopathy

**Fig. 5:**
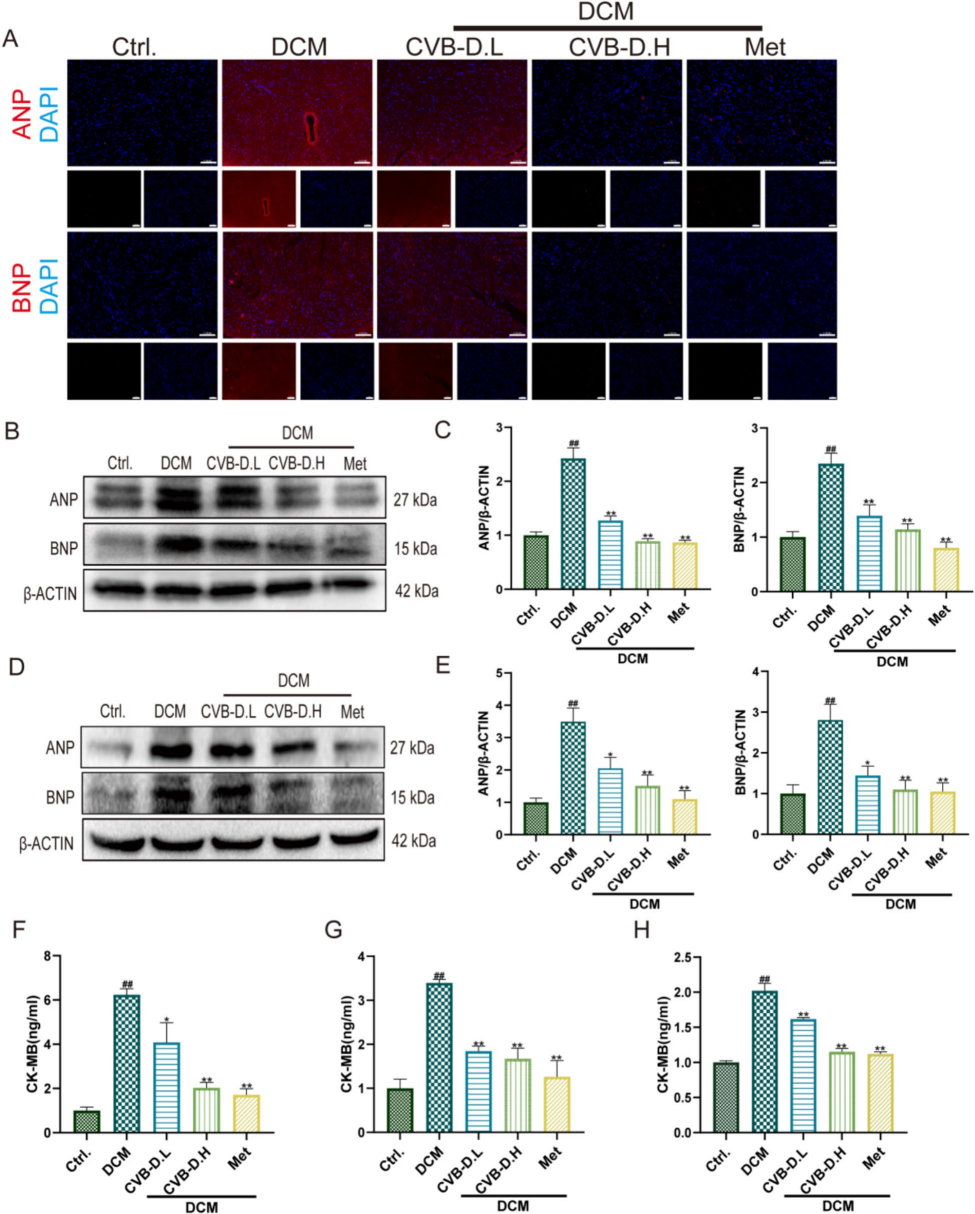
CVB-D attenuates heart failure progression in diabetic dilated cardiomyopathy. **A** Representative immunofluorescence staining showing ANP and BNP expression in myocardial tissues of DCM mice (scale bar = 100 µm). **B**, **C** Western blot analysis and densitometric quantification of ANP and BNP protein levels in cardiac tissues from DCM mice (n = 5). **D**, **E** Western blot analysis and densitometric quantification of ANP and BNP protein levels in NMVMs (n = 5). **F**–**H** ELISA-based measurement of CK-MB levels in mouse serum, cardiac tissues, and NMVMs (n = 6). ^#^*p* < 0.05, ^##^*p* < 0.01 vs. the control group; ^*^p < 0.05, ^**^p < 0.01 vs. the model group

To evaluate the effects of CVB-D on heart failure–related indicators, IF staining and western blotting were performed in both in vivo and in vitro settings. The results showed that ANP and BNP expression was significantly upregulated in the DCM group in mouse cardiac tissues, as evidenced by enhanced IF signals and increased protein abundance on immunoblots (Fig. 5A–C). Notably, CVB-D treatment (low and high doses) markedly attenuated the abnormal elevation of ANP/BNP in DCM mice. Consistent with the in vivo findings, CVB-D also significantly reversed the HG/PA-induced upregulation of ANP and BNP proteins in the NMVM model (Fig. 5D, E).

To further assess myocardial injury, creatine kinase-MB (CK-MB) levels were measured using an ELISA kit in mouse serum, cardiac tissues, and NMVMs. CK-MB levels were significantly higher in the model group than in the control group, whereas CVB-D treatment markedly reduced CK-MB levels across these samples (Fig. 5F–H). Collectively, these results indicate that CVB-D suppresses the upregulation of key heart failure markers under diabetic stress and alleviates cardiomyocyte injury.

### JAK1 plays an indispensable role in CVB-D-mediated protection against mitochondrial dysfunction

**Fig. 6:**
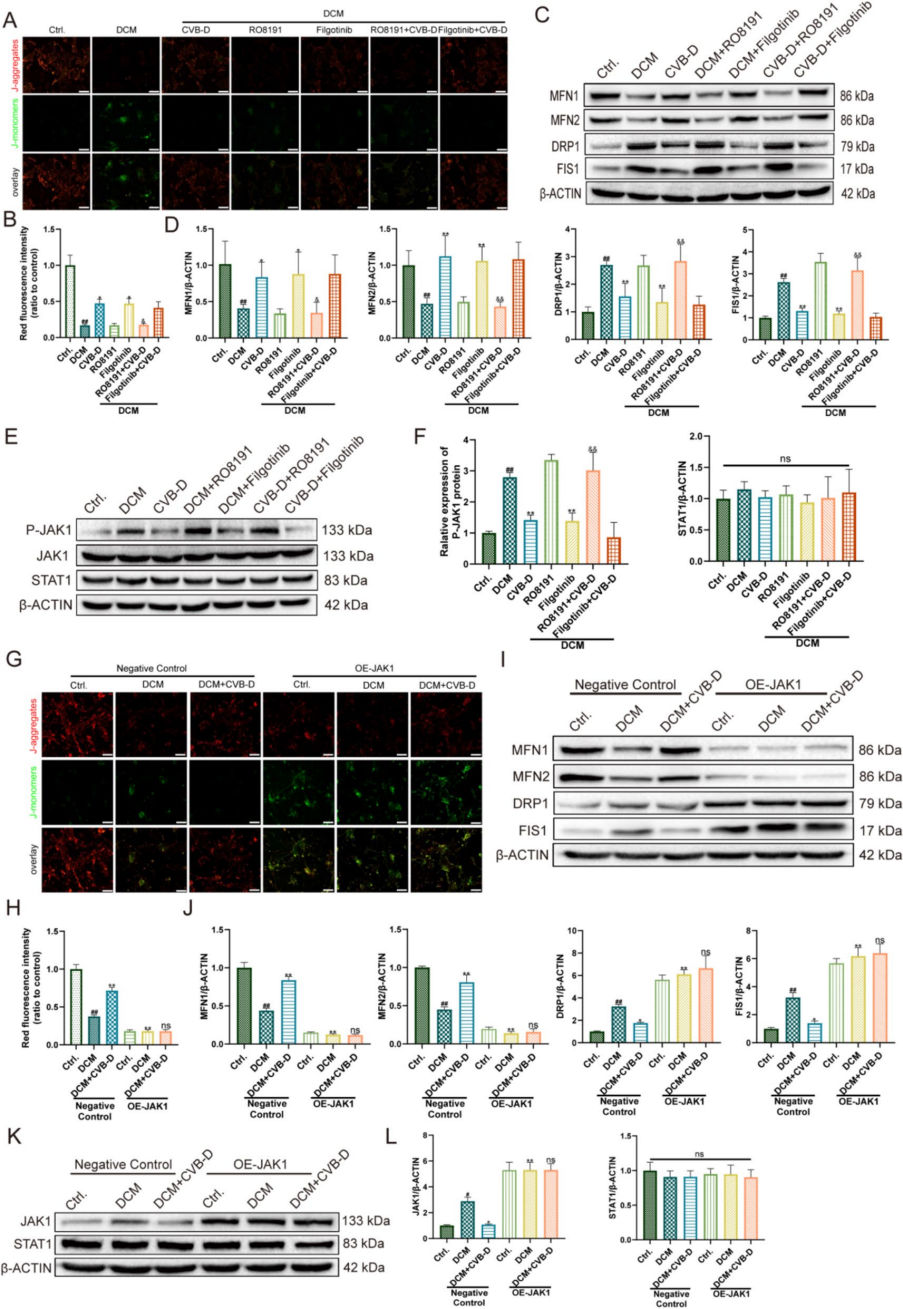
CVB-D ameliorates HG/PA-induced mitochondrial dysfunction by modulating JAK1. **A**, **B** Representative JC-1 staining images and quantitative analysis showing the effects of RO8191 or filgotinib on mitochondrial membrane potential (ΔΨm) in NMVMs (n = 5, scale bar = 50 µm). **C**, **D** Western blot analysis and densitometric quantification of the mitochondrial dynamics–related proteins MFN1, MFN2, DRP1, and FIS1 following RO8191 or filgotinib pretreatment (n = 5). **E**, **F** Western blot analysis and densitometric quantification of p-JAK1, total JAK1, and STAT1 protein levels after RO8191 or filgotinib pretreatment (n = 5). **G**, **H** Representative JC-1 staining images and quantitative analysis showing the effect of JAK1 overexpression (OE-JAK1) on ΔΨm in NMVMs (n = 5, scale bar = 50 µm). **I**, **J** Western blot analysis and densitometric quantification of MFN1, MFN2, DRP1, and FIS1 protein levels following OE-JAK1 treatment (n = 5). **K**, **L** Western blot analysis and densitometric quantification of total JAK1 and STAT1 protein levels following OE-JAK1 treatment (n = 5). ^#^*p* < 0.05, ^##^*p* < 0.01 versus the control group; ^*^p < 0.05, ^**^p < 0.01 versus the model group; ^&^*p* < 0.05, ^&&^*p* < 0.01 versus the CVB-D group

To elucidate the role of JAK1 in the protective effects of CVB-D against HG/PA-induced mitochondrial dysfunction, we used the JAK1 agonist RO8191 (2 μmol/L) and the inhibitor Filgotinib (10 nmol/L) to determine whether JAK1 is a crucial mediator of CVB-D protection. After CVB-D preconditioning of NMVMs in vitro, we measured the JC-1 and other mitochondrial function markers. We found that RO8191 decreased ΔΨm (Fig. 6A, B), increased ROS levels (Fig. S4A). Mitochondrial dynamics are regulated by MFN1, MFN2, DRP1, and FIS1 proteins. RO8191 downregulated the expression of mitochondrial function-related proteins such as MFN1 and MFN2. The expression of mitochondrial function-related proteins DRP1 and FIS1 was also upregulated (Fig. 6C, D). RO8191 abolished the protective effects of CVB-D against HG/PA-induced mitochondrial dysfunction. In contrast, Filgotinib treatment increased ΔΨm (Fig. 6A, B), decreased ROS levels (Fig. S4A), downregulated the expression of mitochondrial fission-related proteins DRP1 and FIS1 and upregulated the expression of mitochondrial function-related proteins MFN1, and MFN2 (Fig. 6C, D). There were no significant differences observed in NMVMs preconditioned with a combination of CVB-D and Filgotinib compared with those preconditioned with Filgotinib alone. Western blotting analysis revealed no significant differences in the total protein levels of JAK1 and STAT1, while P-JAK1 showed marked upregulation after RO8191 treatment and downregulation following Filgotinib administration (Fig. 6E, F), supporting the notion that the observed phenotypes are closely linked to the phosphorylation-dependent activation state of JAK1.

To further establish causality, JAK1 overexpression (OE-JAK1) was used to interrogate the impact of JAK1 activation on mitochondrial homeostasis. Western blotting confirmed efficient JAK1 overexpression (Fig. S4B). OE-JAK1 significantly decreased ΔΨm (Fig. 6G, H) and increased ROS levels (Fig. S4C), accompanied by reduced MFN1/MFN2 and increased DRP1/FIS1 expression (Fig. 6I, J). Under OE-JAK1 conditions, CVB-D-mediated improvements in these parameters were markedly attenuated, whereas total STAT1 levels remained unchanged (Fig. 6K, L). Collectively, these data indicate that JAK1 activation is a critical driver of HG/PA-induced mitochondrial dysfunction and diminishes the protective effects of CVB-D, whereas pharmacological inhibition of JAK1 promotes restoration of mitochondrial homeostasis and supports CVB-D-mediated mitochondrial protection.

### JAK1 knockdown potentiates CVB-D-mediated protection against mitochondrial dysfunction in vitro and in vivo

**Fig. 7:**
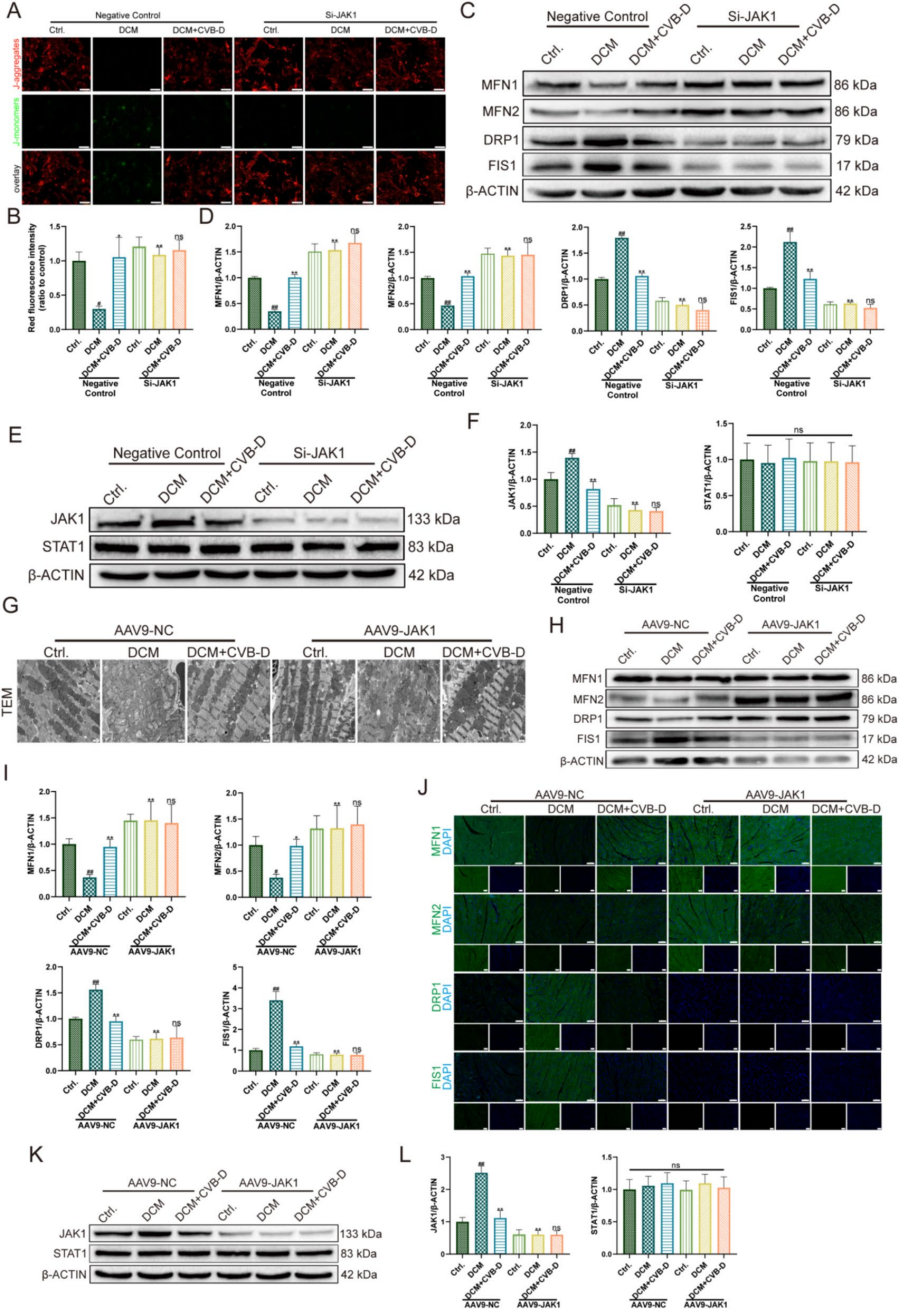
JAK1 knockdown potentiates CVB-D-mediated protection against mitochondrial dysfunction In vitro and In vivo. **A**, **B** Representative JC-1 staining images and quantitative analysis showing that Si-JAK1 treatment significantly improves mitochondrial membrane potential (ΔΨm) in NMVMs (n = 5, scale bar = 50 µm). **C**, **D** Western blot analysis and densitometric quantification of mitochondrial dynamics–related proteins after Si-JAK1 treatment, showing upregulation of the fusion proteins MFN1 and MFN2 and downregulation of the fission-related proteins DRP1 and FIS1 (n = 5). **E**, **F** Western blot analysis and densitometric quantification of total JAK1 and STAT1 protein levels after Si-JAK1 treatment (n = 5). **G** Representative TEM images showing mitochondrial ultrastructural changes in mouse hearts after AAV9-GP-JAK1 injection (scale bar = 1 µm). **H**, **I** Western blot analysis and densitometric quantification of MFN1, MFN2, DRP1, and FIS1 protein levels in cardiac tissues from AAV9-GP-JAK1–transduced mice (n = 5). **J** Representative immunofluorescence images showing MFN1, MFN2, DRP1, and FIS1 expression in cardiac tissues from AAV9-GP-JAK1–transduced mice (scale bar = 100 µm). **K**, **L** Western blot analysis and densitometric quantification of total JAK1 and STAT1 protein levels after AAV9-GP-JAK1 treatment (n = 5). ^#^*p* < 0.05, ^##^*p* < 0.01 versus the control group; ^*^p < 0.05, ^**^p < 0.01 versus the model group

To further clarify the role of JAK1 in mitochondrial injury and the protective effects of CVB-D, we conducted genetic loss-of-function experiments targeting JAK1 in vitro and in vivo. In NMVMs, several siRNAs were screened and knockdown efficiency was verified by western blotting; among them, Si-JAK1-3306 produced the most pronounced reduction in JAK1 protein abundance (Fig. S4D). Functionally, JAK1 knockdown significantly increased ΔΨm, as reflected by an elevated JC-1 red/green fluorescence ratio (Fig. 7A, B), and attenuated oxidative stress, evidenced by reduced ROS signals and further confirmed by flow cytometry (Fig. S4E). Additionally, we employed the Mito-ROS staining method to detect mitochondrial oxidative stress levels. The results were consistent with those of ROS (Fig. S4G). Moreover, JAK1 silencing restored mitochondrial dynamics homeostasis by upregulating the fusion proteins MFN1/MFN2 and downregulating the fission-related proteins DRP1/FIS1 (Fig. 7C, D). In parallel, western blotting indicated that total STAT1 protein levels were unchanged, whereas JAK1 was markedly reduced; under JAK1-deficient conditions, CVB-D treatment did not further alter total JAK1 or STAT1 abundance (Fig. 7E, F).

To validate the requirement of JAK1 for mitochondrial regulation in vivo, we generated a cardiomyocyte-targeted JAK1 knockdown model via intravenous administration of adeno-associated virus serotype 9 (AAV9) vectors expressing shRNA against JAK1 (AAV9-GP-1-JAK1; 5′-ctgtcctgatgaggtttat-3′) or a negative-control shRNA (AAV9-GP-1-NC; 5′-actaccgttgttataggtg-3′) for 4 weeks. Robust myocardial transduction was confirmed by SpRed fluorescence (Fig. S4F). TEM revealed improved mitochondrial morphology and cristae organization (Fig. 7G). Consistently, western blotting (Fig. 7H, I) and IF (Fig. 7J) analyses demonstrated normalization of mitochondrial dynamics markers, characterized by increased MFN1/MFN2 and decreased DRP1/FIS1 expression. JAK1 was effectively downregulated in DCM hearts, whereas total STAT1 remained unchanged (Fig. 7K, L).

Collectively, these data indicate that JAK1 deficiency mitigates mitochondrial dysfunction by suppressing oxidative stress and restoring the balance between mitochondrial fusion and fission, supporting JAK1 as a critical regulator of mitochondrial injury in DCM and a key mediator of CVB-D-associated mitochondrial protection.

### JAK1 is a central mediator of CVB-D-driven protection against heart failure

To further delineate the role of JAK1 in CVB-D-mediated protection against diabetic stress–associated heart failure progression, we pharmacologically and genetically modulated JAK1 in HG/PA-treated NMVMs and DCM mice, and quantitatively assessed established markers of heart failure and myocardial injury.

**Fig. 8:**
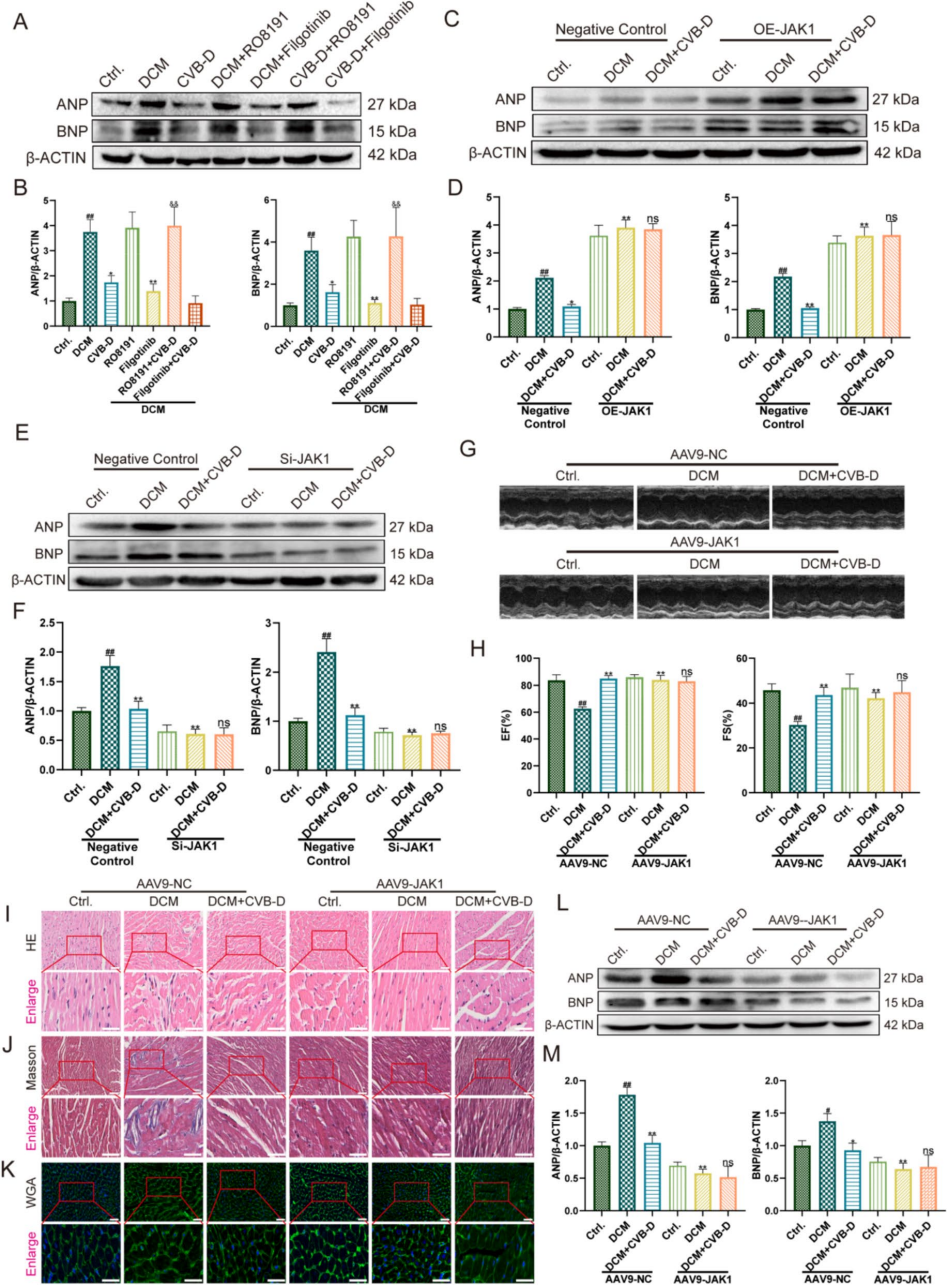
JAK1 is a central mediator of CVB-D-driven protection against heart failure. **A**, **B** Western blot analysis and densitometric quantification of ANP and BNP protein levels in NMVMs after pretreatment with RO8191 or filgotinib (n = 5). **C**, **D** Western blot analysis and densitometric quantification of ANP and BNP expression in NMVMs after OE-JAK1 treatment (n = 5). **E**, **F** Western blot analysis and densitometric quantification of ANP and BNP expression in NMVMs after Si-JAK1 treatment (n = 5). **G** Representative M-mode echocardiographic images for each group. (H) Quantitative analysis of EF% and FS% (n = 6). **I**–**K** Representative images of H&E, Masson’s trichrome, and WGA staining in heart tissues (scale bar = 50 µm). **L**, **M** Western blot analysis and densitometric quantification of ANP and BNP protein levels after AAV-GP-JAK1 treatment (n = 5). ^#^*p* < 0.05, ^##^*p* < 0.01 versus. the control group; ^*^p < 0.05, ^**^p < 0.01 versus the model group; ^&^*p* < 0.05, ^&&^*p* < 0.01 versus the CVB-D group

In vitro, western blotting demonstrated that HG/PA markedly increased ANP and BNP expression, whereas CVB-D significantly attenuated this induction. Pharmacological activation of JAK1 with RO8191 further elevated ANP/BNP levels and largely abrogated the suppressive effects of CVB-D on these markers; conversely, the JAK1 inhibitor filgotinib significantly reduced ANP/BNP expression (Fig. 8A, B). Consistently, JAK1 overexpression (OE-JAK1) increased ANP/BNP abundance and weakened CVB-D-mediated suppression (Fig. 8C, D). In contrast, JAK1 knockdown (Si-JAK1) produced a greater reduction in ANP/BNP than CVB-D alone, and under conditions of effective JAK1 suppression, co-treatment with CVB-D did not yield a further detectable decrease, indicating that JAK1 inhibition is a dominant driver of ANP/BNP downregulation and limits the additivity of CVB-D (Fig. 8E, F).

To assess myocardial injury, we further quantified CK-MB. Relative to controls, CK-MB levels were significantly elevated in the model group, consistent with aggravated myocardial injury, and were markedly reduced by CVB-D. RO8191 further increased CK-MB and offset the CK-MB-lowering effect of CVB-D, whereas filgotinib decreased CK-MB in a manner concordant with CVB-D (Fig. S5A). Similarly, OE-JAK1 increased CK-MB and attenuated the reduction induced by CVB-D (Fig. S5B), whereas Si-JAK1 significantly reduced CK-MB, with no additional substantial improvement upon co-treatment with CVB-D (Fig. S5C). Collectively, these data indicate that JAK1 activation exacerbates myocardial injury, whereas JAK1 inhibition alleviates injury and is permissive for CVB-D-mediated cardioprotection.

In vivo, we generated cardiac JAK1 intervention models by intravenous administration of AAV9 vectors (AAV9-NC and AAV9-mediated JAK1 suppression, denoted as AAV9-JAK1). Echocardiography showed that, on the AAV9-NC background, CVB-D significantly improved EF% and FS% in DCM mice. By contrast, under AAV9-mediated JAK1 suppression, cardiac function was already markedly improved and approached control levels, and co-administration of CVB-D did not provide additional significant benefit, indicating that JAK1 inhibition alone confers substantial functional improvement and constrains further gain from CVB-D (Fig. 8G, H). Histological analyses supported these findings, showing that JAK1 suppression mitigated myocardial injury, as evidenced by reduced cardiomyocyte hypertrophy and improved myocardial architecture on H&E/WGA staining and decreased collagen deposition on Masson’s trichrome staining (Fig. 8I–K). Concordantly, western blotting (Fig. 8L and M) and IF (Fig. S5E) analyses revealed reduced ANP/BNP expression in cardiac tissues. In addition, lipid profiling showed increased TC, LDL-C, and TG and decreased HDL-C in DCM mice; CVB-D partially normalized these abnormalities. Under JAK1 suppression, these metabolic derangements were also substantially improved, with limited additional benefit from CVB-D (Fig. S5F-I). Finally, In vivo measurements confirmed that AAV9-mediated JAK1 suppression significantly reduced DCM-associated elevation of CK-MB, consistent with the direction of CVB-D effects (Fig. S5D).

Collectively, these in vitro and in vivo results demonstrate that JAK1 activation promotes upregulation of heart failure markers (ANP/BNP) and the myocardial injury marker CK-MB, thereby weakening CVB-D-mediated cardioprotection. Conversely, JAK1 inhibition/knockdown markedly improves cardiac function and pathological remodeling and reduces ANP/BNP and CK-MB. Because the magnitude of benefit conferred by JAK1 suppression exceeds that of CVB-D alone, the incremental effect of CVB-D becomes limited or non-significant under JAK1-suppressed conditions. These findings support JAK1 as a key regulatory node underlying the cardioprotective actions of CVB-D.

### CVB-D exerts cardioprotection by inhibiting JAK1-mediated STAT1 phosphorylation

**Fig. 9:**
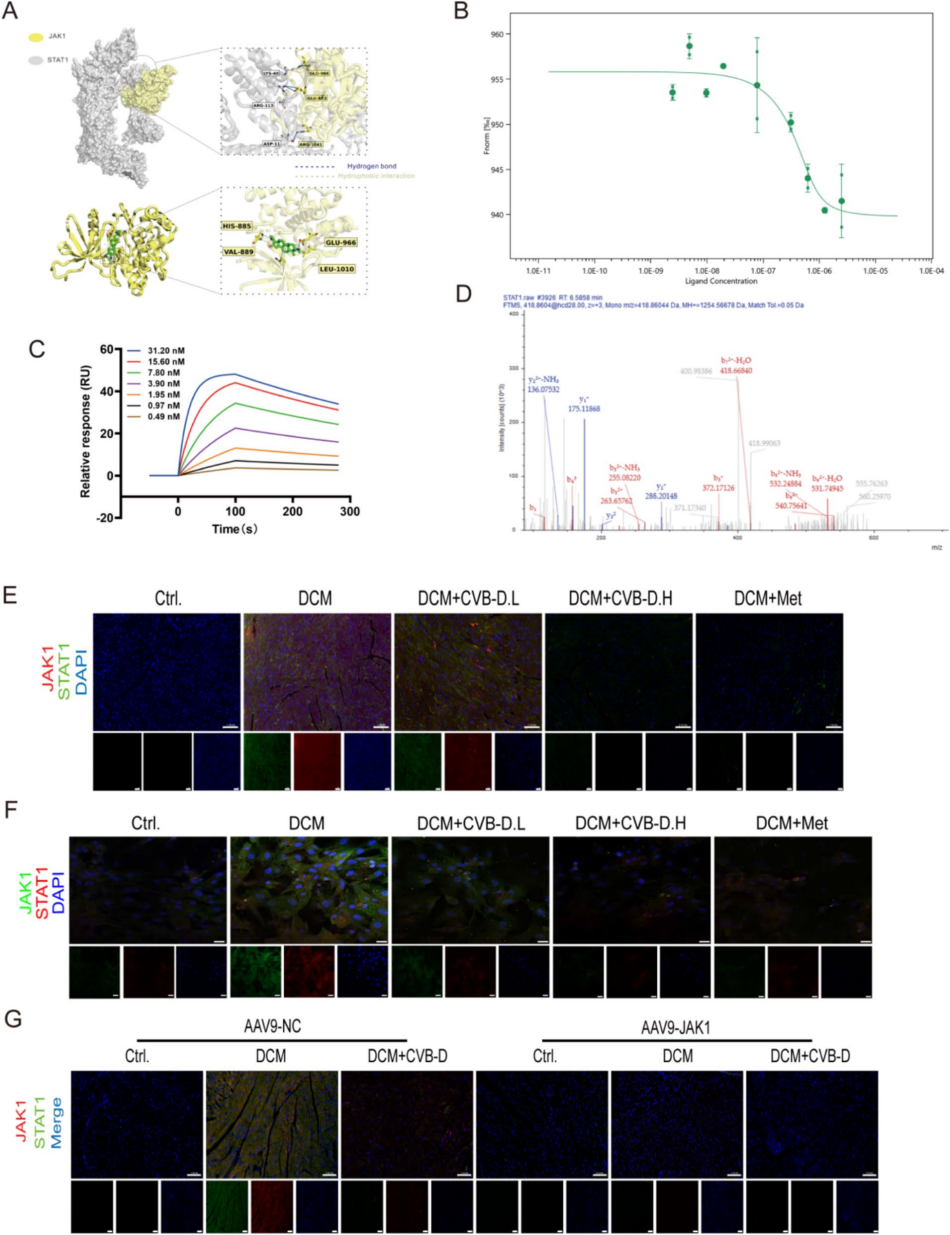
CVB-D exerts cardioprotection by inhibiting JAK1-mediated STAT1 phosphorylation. **A** Molecular docking of CVB-D to JAK1 protein. **A** Molecular docking between JAK1 and STAT1 protein. **B** MST analysis of the JAK1 and STAT1 interactions. **C** Binding affinity analysis of JAK1 and STAT1 determined by SPR. **D** LC-MS/MS was used to analyze the acetylation sites of STAT1. **E**–**G** IF images of JAK1 and STAT1 colocalization in NMVMs (scale bar = 100 µm)

To determine whether CVB-D directly targets JAK1 and modulates the JAK1-STAT1 axis, we combined in silico modeling with biophysical and biochemical validation. Molecular docking predicted that CVB-D occupies the JAK1 catalytic pocket with a binding energy of approximately − 7.3 kcal/mol. Structural analyses suggested that CVB-D is stabilized by a hydrogen bond with HIS-885 and hydrophobic contacts with VAL-889 and LEU-1010, and that the predicted ligand-binding region is proximal to functionally important residues including GLU-966 (Fig. 9A). Protein–protein docking further indicated strong structural complementarity between JAK1 and STAT1 (ZDOCK score 1012.32), with the binding interface supported by a hydrogen-bond network involving STAT1 residues LYS-40, ARG-113, and ASP-11 and JAK1 residues GLU-966, GLU-883, and ARG-1041. Notably, GLU-966 appears to serve as an anchoring residue for STAT1 binding and lies near the predicted CVB-D binding site, raising the possibility that CVB-D may impede JAK1-STAT1 complex formation via competitive occupancy and/or steric interference (Fig. 9A).

Experimentally, MST confirmed a specific interaction between JAK1 and STAT1, with a dissociation constant of KD ≈ 2.52 μM (Fig. 9B). SPR further validated their direct binding and yielded kinetic parameters consistent with high-affinity association (ka = 1.73 × 10⁶ M⁻^1^·s⁻^1^, kd = 1.94 × 10⁻^3^ s⁻^1^, KD = 1.12 × 10⁻⁹ M) (Fig. 9C). Because CVB-D-mediated protection is linked to STAT1 dephosphorylation but the relevant sites have not been defined, LC–MS/MS analysis was performed to map putative STAT1 phosphorylation sites, identifying T598 and T699 as candidate regulatory residues (Fig. 9D).

Mechanistically, IF co-localization analyses demonstrated that JAK1 and STAT1 co-localize in cardiomyocytes. Co-localization was increased in the DCM group relative to controls and was markedly restored toward baseline following CVB-D treatment (Fig. 9E, F). Consistently, in an AAV9-mediated cardiac JAK1 knockdown model, JAK1 signals and its co-localization with STAT1 were further reduced, supporting the notion that reduced JAK1 abundance weakens JAK1-STAT1 association in vivo (Fig. 9G). Collectively, these data support a model in which CVB-D binds to JAK1 and disrupts JAK1-STAT1 interaction, thereby dampening JAK1-STAT1-dependent signaling and ultimately attenuating DCM progression.

## Discussion

DCM is a significant cardiovascular complication of diabetes, independent of major confounding cardiac conditions such as coronary artery disease and hypertension [27]. CVB-D is a naturally occurring bioactive alkaloid isolated from *Buxus microphylla* and has been widely applied in the management of cardiovascular and cerebrovascular disorders. Notably, previous studies have reported that CVB-D-triggered mitophagy can enhance apoptotic signaling by exacerbating mitochondrial dysfunction [28]. However, the precise mechanisms by which CVB-D exerts therapeutic effects in DCM remain incompletely defined, which hampers its pharmacological development and potential clinical translation. To determine whether CVB-D can ameliorate DCM and to elucidate the underlying mechanism, we conducted the present study. Our results show that CVB-D alleviates DCM-associated mitochondrial dysfunction, thereby delaying the progression of HF in both in vitro and in vivo models. In this study, we identified JAK1 as a direct binding target of STAT1. Moreover, we found that CVB-D suppresses JAK1 expression, which is accompanied by reduced STAT1 phosphorylation and improved mitochondrial function. Collectively, these findings provide new insights into the molecular target(s) and pharmacological mechanisms underlying the protective effects of CVB-D in DCM.

In cardiomyocytes, mitochondrial oxidative metabolism provides the vast majority of this energy, accounting for approximately 95% of total ATP production [29]. Mitochondrial bioenergetic capacity is tightly coupled to organelle morphology, which is continuously remodeled by mitochondrial fusion and fission dynamics to maintain an architecture compatible with cellular energy demands [30]. TEM was used to examine mitochondrial ultrastructure in mouse hearts to determine whether the beneficial effects of CVB-D in DCM are associated with mitochondrial protection. In the DCM group, mitochondria exhibited disrupted cristae, marked swelling, and pronounced fragmentation. In contrast, CVB-D treatment significantly improved mitochondrial morphology. In vivo, DCM was accompanied by reduced ATP production and increased L-LDH activity. Western blot analysis further showed decreased levels of the mitochondrial fusion proteins MFN1 and MFN2 and increased levels of the fission-related proteins DRP1 and FIS1; notably, these alterations were reversed by CVB-D treatment (Fig. 3). Consistent results were obtained in NMVMs (Fig. 4). Collectively, these findings suggest that the therapeutic effects of CVB-D in DCM are at least partly mediated through preservation of mitochondrial integrity and dynamics.

Impaired mitochondrial bioenergetics represents both a hallmark of HF and a pathogenic driver that exacerbates disease progression. Mitochondrial dysfunction accelerates ROS production and prompts cytochrome c release into the cytosol, thereby promoting programmed cell death, cardiomyocyte injury, and the advancement of HF [31]. Our results showed that the expression of heart failure-associated proteins was increased in the DCM group, accompanied by elevated CK-MB levels. In contrast, CVB-D treatment effectively attenuated the progression of HF (Fig. 5). These findings support the hypothesis that CVB-D protects cardiomyocytes against HF, at least in part, by improving mitochondrial function. Nevertheless, the precise mechanisms by which CVB-D enhances mitochondrial function remain to be fully elucidated and warrant further investigation.

Previous studies have shown that CVB-D attenuates lipopolysaccharide-induced inflammatory responses in murine macrophages In vitro, an effect associated with inhibition of JAK-STAT signaling [32]. To elucidate the molecular basis by which CVB-D preserves mitochondrial function and delays cardiomyocyte failure, we further interrogated the role of JAK1 in our models. In NMVMs, pharmacological modulation of JAK1 produced a coherent pattern: the JAK1 inhibitor filgotinib markedly mitigated mitochondrial injury and improved heart failure-related phenotypes, whereas the JAK1 activator RO8191 attenuated or reversed the protective effects of CVB-D (Figs. 6 and 8). Consistently, enforced JAK1 expression using PEX-3 exacerbated mitochondrial damage and accelerated the progression of cardiomyocyte failure, thereby counteracting CVB-D-mediated protection of mitochondrial and cardiac function (Figs. 6 and 8). Moreover, complementary JAK1 loss-of-function approaches (si-JAK1 in NMVMs and AAV9-GP-JAK1-based manipulation in vivo) further support that the protective effects of CVB-D in DCM are dependent on JAK1 expression (Figs. 7 and 8). Collectively, these data indicate that CVB-D confers cardioprotection by suppressing JAK1 and consequently limiting STAT1 phosphorylation/activation, thereby maintaining mitochondrial homeostasis and slowing DCM-associated HF progression.

STAT1 is a transcription factor that modulates gene programs controlling cell-cycle progression, survival pathways, and immune responses. Altered mitochondrial STAT1 content has been associated with impaired mitochondrial function, including disruptions in mitochondrial biogenesis and the maintenance of mitochondrial homeostasis [33]. Next, we focused on STAT1, a key downstream effector of JAK1, which may serve as a pivotal regulator linking stress signaling to mitochondrial homeostasis. Previous studies have indicated that interleukin-6 (IL-6) can impair mitochondrial function via activation of the JAK1–STAT1/STAT3 signaling axis [34]. Collectively, these findings suggest that STAT1 is not only a canonical transcriptional node in inflammatory and stress responses but may also be tightly coupled to mitochondrial dynamics (particularly DRP1-dependent fission) and cell fate regulation. However, in the context of our study, the key phosphorylation sites of STAT1 in DCM remain undefined, and direct evidence is still lacking as to whether and how STAT1 phosphorylation drives mitochondrial dysfunction and dysregulated mitochondrial dynamics. Therefore, future studies should systematically identify the critical STAT1 phosphorylation sites and delineate the molecular pathways through which STAT1 modulates mitochondrial homeostasis, thereby providing stronger causal support for the mechanism by which CVB-D ameliorates DCM via the JAK1-STAT1 axis.

IF co-localization analyses demonstrated a robust spatial association between JAK1 and STAT1 in cardiomyocytes, which was enhanced under DCM conditions and markedly restored toward baseline following CVB-D treatment; consistent with this, AAV9-mediated cardiac JAK1 knockdown further reduced JAK1 signals and JAK1-STAT1 co-localization, suggesting that JAK1 abundance is a key determinant of complex formation in vivo. Supported by integrated computational and biophysical evidence, molecular docking predicted that CVB-D may occupy the JAK1 catalytic pocket and potentially interfere with the JAK1-STAT1 binding interface, whereas MST and SPR verified direct JAK1-STAT1 binding with high affinity. Functionally, diabetic stress (DCM in vivo and HG/PA exposure in vitro) induced mitochondrial ultrastructural disruption, decreased ATP production, and increased L-LDH activity, along with a shift in mitochondrial dynamics toward a fission-dominant profile (MFN1/MFN2 downregulation with DRP1/FIS1 upregulation). Notably, CVB-D reversed these alterations in both in vivo and in vitro settings and significantly restored mitochondrial respiratory function. Mechanistically, CVB-D suppressed p-JAK1 without markedly changing total JAK1-STAT1 levels and restored mitochondrial homeostasis in the context of STAT1-DRP1 interaction, ultimately attenuating HF markers (ANP/BNP) and myocardial injury (CK-MB); convergent pharmacological and genetic manipulation of JAK1 (activation, inhibition, overexpression, and knockdown) produced directionally consistent phenotypes, collectively establishing the JAK1-STAT1 axis as a central node mediating CVB-D cardioprotection in diabetic DCM.

Further research is required to address the following and other emerging issues: (1) Although LC-MS/MS detection revealed phosphorylation modifications at the T598/T699 sites of STAT1 protein, the direct functional contributions of these sites still need to be further validated through site-directed mutagenesis and backfilling experiments. (2) The direct molecular mechanism by which CVB-D regulates JAK1 remains to be elucidated. (3) The molecular mechanism by which STAT1/p-STAT1 regulates mitochondrial homeostasis has not been fully elucidated, and its role may not be entirely dependent on DRP1-mediated mitochondrial fission. Despite these limitations, this study demonstrates that CVB-D enhances mitochondrial function through the JAK1-STAT1 axis, thereby exerting a protective effect on heart failure. This reveals a unique mechanism by which CVB-D provides mitochondrial protection, which may offer new insights for screening potential therapeutic candidates for dilated cardiomyopathy.

## Conclusions

Taken together, our data indicate that CVB-D presents cardioprotection in diabetic cardiomyopathy by restoring mitochondrial homeostasis and ameliorating heart failure–related phenotypes in both PA/HG-challenged NMVMs and HFD/STZ-induced DCM mice. Mechanistically, CVB-D could directly target JAK1 and attenuate aberrant JAK1-STAT1 signaling, thereby mitigating mitochondrial ultrastructural damage, rebalancing mitochondrial fusion–fission dynamics, and improving cardiomyocyte function. Collectively, these findings delineate a JAK1/STAT1-dependent, mitochondria-centered mode of action for CVB-D and support its further development as a therapeutic candidate for DCM-associated heart failure.

## Supplementary Information

Additional file 1.

## Data Availability

The data in this study are available from the corresponding author upon reasonable request.

## Abbreviations

ANP: Atrial natriuretic peptides
AUC: Areas under the curve
BCA: Bicinchoninic acid
BNP: B-type natriuretic peptide
BSA: Bovine serum albumin
CVB-D: Cyclovirobuxine D
cTnT: Cardiomytroponin T
DAPI: 4', 6-diamidino-2-phenylindole
DM: Diabetes mullins
DMSO: Dimethyl sulfoxide
DRP1: Dynamin-related protein
DCM: Diabetic cardiomyopathy
EF%: Ejection fraction
FS%: Fractional shortening
FBG: Fasting blood glucose
FBS: Fetal bovine serum
FIS1: Fission 1
HG/PA: High glucose/Palmitic acid
HFD: High-fat diet
H&E: Hematoxylin-eosin
HOMA-IR: Homeostasis model assessment-Insulin resistance
IF: Immunofluorescence
IP: Immunoprecipitation
IPGTT: Intraperitoneal glucose tolerance test
L-LDH: L-Lactate dehydrogenase
MFN1/2: Mitofusin1/2
MMP: Mitochondrial membrane potential
MTT: 3-(4,5-dimethyl-2- thiazolyl)-2,5-diphenyl-2-H-tetrazolium bromide
MST: Microscale thermophoresis
NMVMs: Primary neonatal mouse ventricular myocytes
OCR: Oxygen consumption rate
OGTT: Oral glucose tolerance test
PBS: Phosphate-buffered saline
PMSF: Phenylmethyl sulfonyl fluoride
PVDF: Polyvinylidene difluoride
ROS: Reactive oxygen species
SDS-PAGE: SDS-polyacrylamide gel electrophoresis
SPR: Surface plasmon resonance
STZ: Streptozotocin
T2DM: Type 2 diabetes mellitus
TEM: Transmission electron microscopy
TBST: Tris buffered saline with Tween-20
WGA: Wheat germ agglutinin
β-actin: Beta-muscle actin
