# Supplementary material for: CVB-D attenuates experimental diabetic cardiomyopathy by alleviating mitochondrial dysfunction via the JAK1-STAT1 signaling axis in vivo and in vitro
Source: Chin Med. 2026 May 13;21:131. doi: 10.1186/s13020-026-01411-2 (PMC13169674; doi:10.1186/s13020-026-01411-2)
Supplement: Supplementary file 1 — Additional file 1. [file 13020_2026_1411_MOESM1_ESM.docx]

**Supplementary Material**


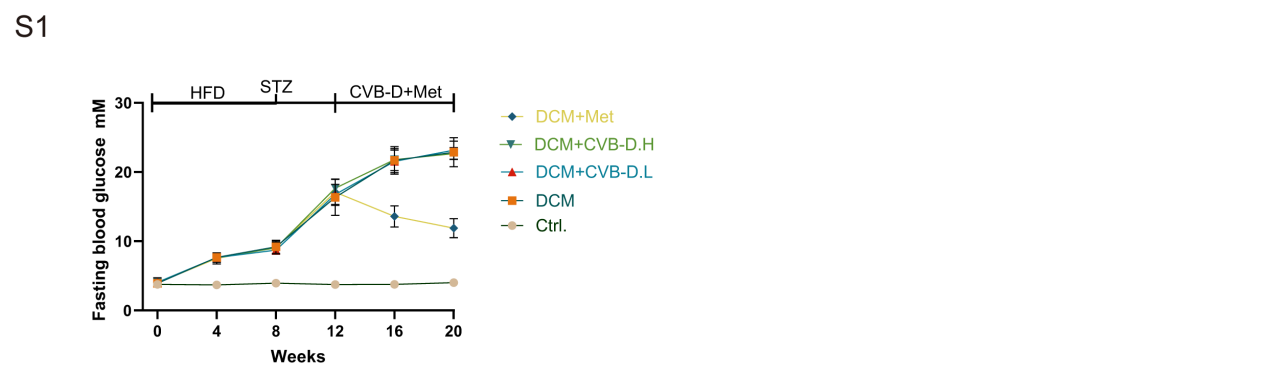


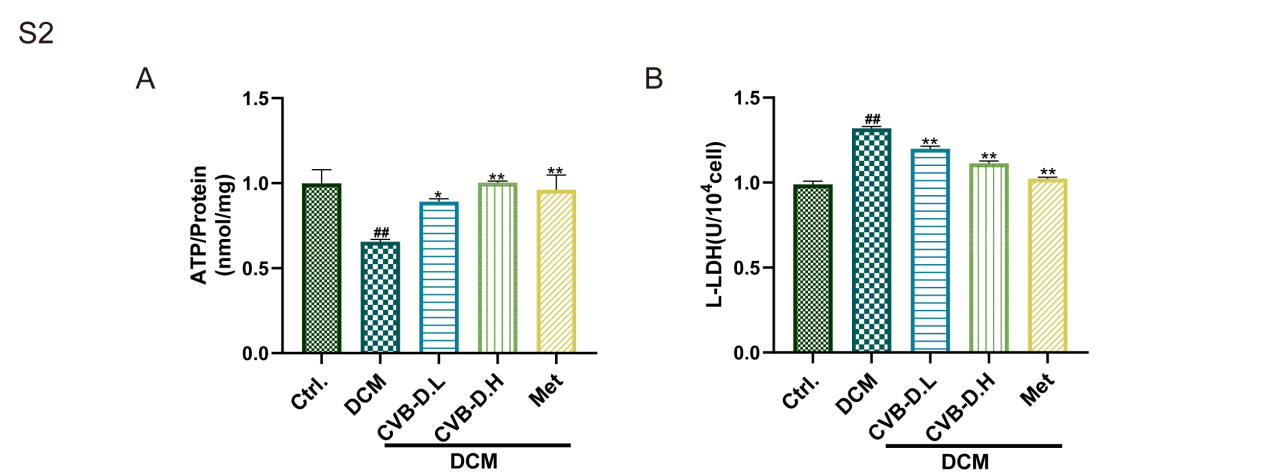


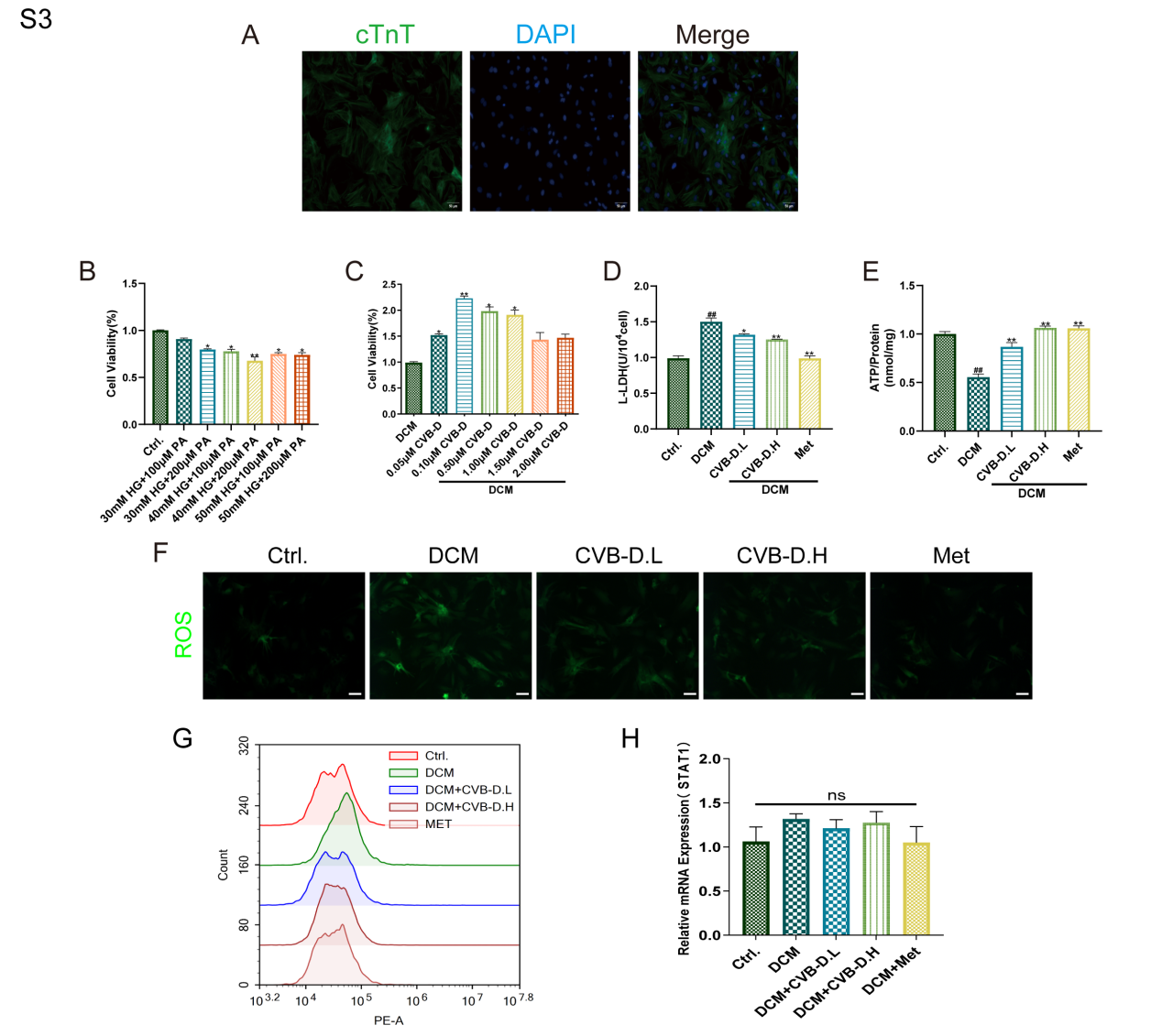

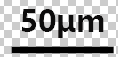

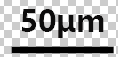

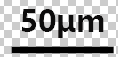

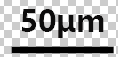

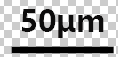

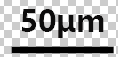

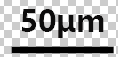

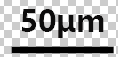


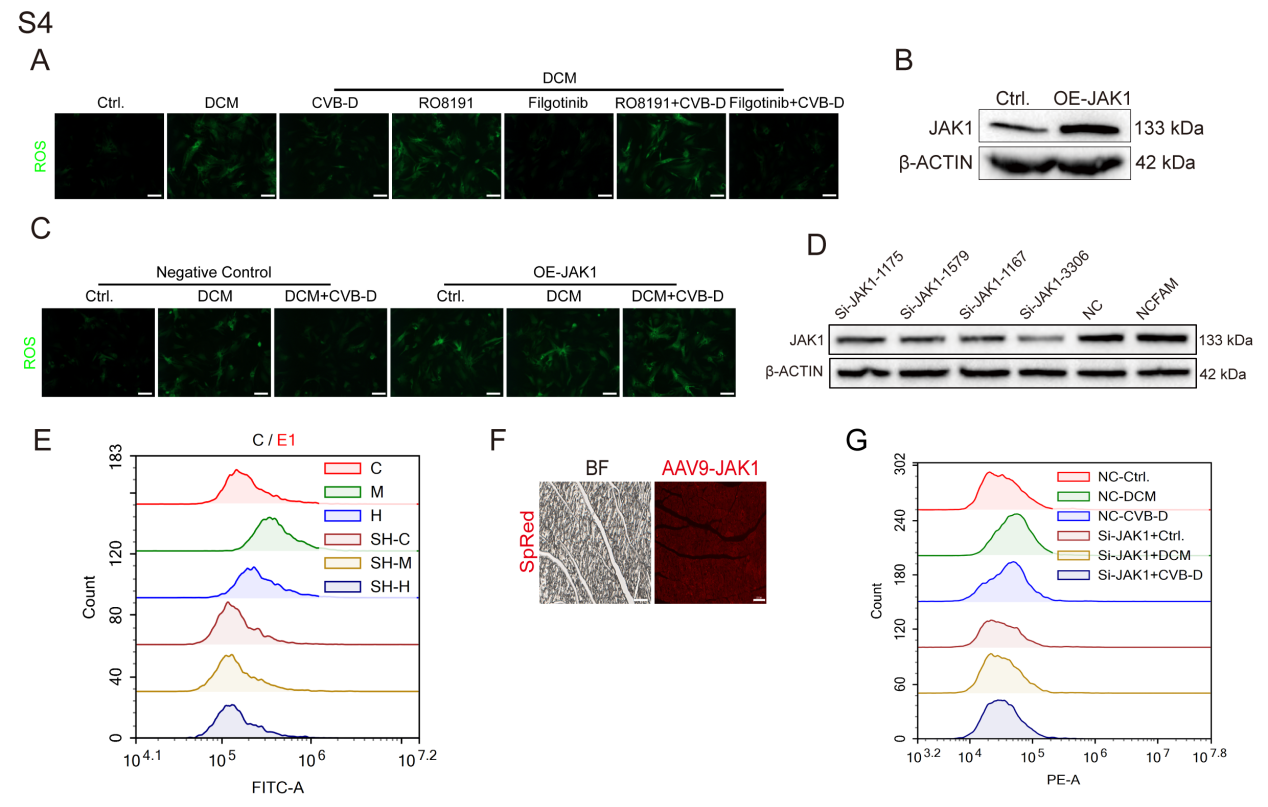

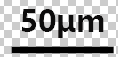

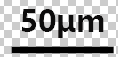

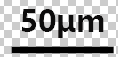

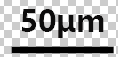

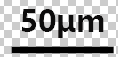

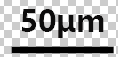

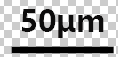

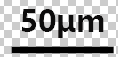

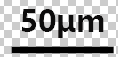

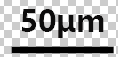

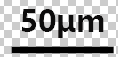

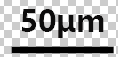

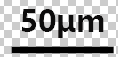

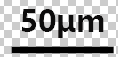

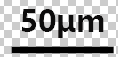


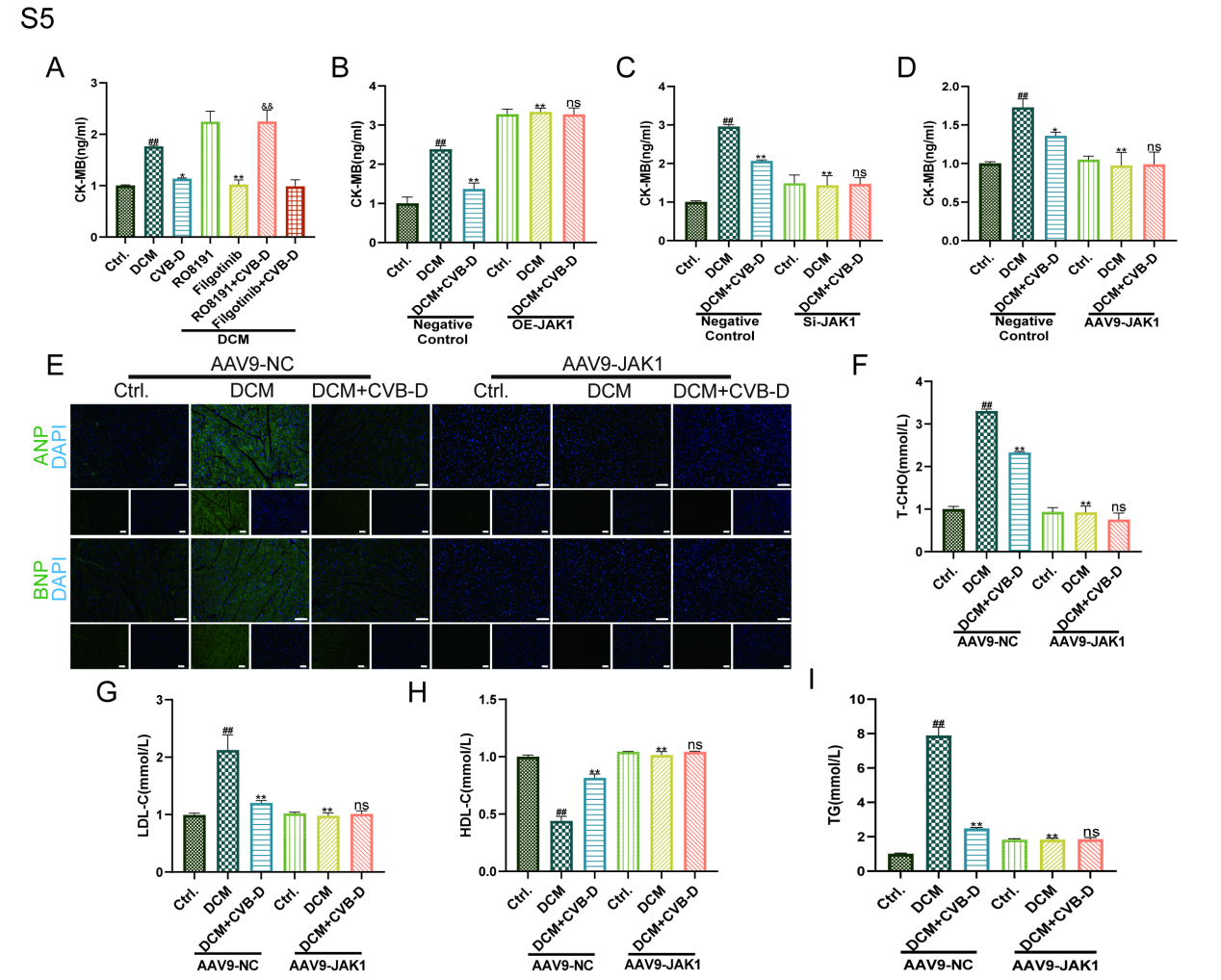

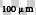

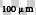

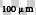

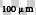

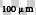

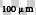

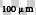

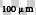

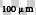

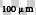

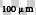

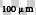


(S1) Body weight of mice (n = 6). ^#^*p* < 0.05, ^##^*p* < 0.01 versus the control group; ^*^*p* < 0.05, ^**^*p* < 0.01 versus the model group.

(S2 A) ATP content of mouse cardiac tissues (n = 3). (S2 B) L-LDH activity (n = 3). ^#^*p* < 0.05, ^##^*p* < 0.01 versus the control group; ^*^*p* < 0.05, ^**^*p* < 0.01 versus the model group.

(S3 A) Representative images of immunofluorescence dyeing of cTnT in the NMVMs (scale bar = 50 µm). (S3 B) and (S3 C) Cell viability of each group was determined by MTT assay (n = 3). (S3 D) ATP content of mouse cardiac tissues (n = 3). (S3 E) L-LDH activity (n = 3). (S3 F) ROX fluorescence staining (scale bar = 50 µm). (S3 G) Flow cytometry was used to examine the MitoROS of NMVMs (n = 3). (S3 H) qRT‒PCR analysis of STAT1 mRNA levels in NMVMs (n = 3). ^#^*p* < 0.05, ^##^*p* < 0.01 versus the control group; ^*^*p* < 0.05, ^**^*p* < 0.01 versus the model group.

(S4 A) and (S4 C) ROX fluorescence staining (scale bar = 50 µm). (S4 B) and (S4 D)Western blotting for JAK1 proteins in NMVMs transfected with OE-JAK1 or Si-JAK1 (n = 3). (S4 E) ROS of NMVMs was measured by flow cytometry (n = 3). (S4 F) Images showing SpRed fluorescence in the heart infected with AAV9-JAK1 (scale bar = 50 µm). (S4 G) Flow cytometry was used to examine the MitoROS of NMVMs (n = 3). ^#^*p* < 0.05, ^##^*p* < 0.01 versus the control group; ^*^*p* < 0.05, ^**^*p* < 0.01 versus the model group.

(S5 A), (S5 B), (S5 C) and (S5 D) ELISA-based quantification of CK-MB after pharmacological or genetic modulation of JAK1 (n = 6). (S5 E) Representative immunofluorescence images of ANP and BNP after **AAV-GP-JAK1** treatment (scale bar = 100 µm). (S5 F), (S5 G), (S5 H) and (S5 I) AAV9-JAK1 on the serum levels of low-density lipoprotein (LDL), high-density lipoprotein (HDL), triglycerides (TG) and total cholesterol (TC) (n > 6). ^#^*p* < 0.05, ^##^*p* < 0.01 versus the control group; ^*^*p* < 0.05, ^**^*p* < 0.01 versus the model group.
